# Deep Sequencing Transcriptome Analysis of Murine Wound Healing: Effects of a Multicomponent, Multitarget Natural Product Therapy-Tr14

**DOI:** 10.3389/fmolb.2017.00057

**Published:** 2017-08-17

**Authors:** Georges St. Laurent, Bernd Seilheimer, Michael Tackett, Jianhua Zhou, Dmitry Shtokalo, Yuri Vyatkin, Maxim Ri, Ian Toma, Dan Jones, Timothy A. McCaffrey

**Affiliations:** ^1^St. Laurent Institute Vancouver, WA, United States; ^2^SeqLL, Inc. Woburn, MA, United States; ^3^Biologische Heilmittel Heel GmbH Baden-Baden, Germany; ^4^Nantong University Nantong, China; ^5^A.P.Ershov Institute of Informatics Systems Novosibirsk, Russia; ^6^AcademGene LLC Novosibirsk, Russia; ^7^Division of Genomic Medicine, The George Washington University Washington, DC, United States

**Keywords:** wound healing, RNA-seq, Traumeel (Tr14), multicomponent, multitarget, natural products, TGF-ß1, transcript profiling

## Abstract

Wound healing involves an orchestrated response that engages multiple processes, such as hemostasis, cellular migration, extracellular matrix synthesis, and in particular, inflammation. Using a murine model of cutaneous wound repair, the transcriptome was mapped from 12 h to 8 days post-injury, and in response to a multicomponent, multi-target natural product, Tr14. Using single-molecule RNA sequencing (RNA-seq), there were clear temporal changes in known transcripts related to wound healing pathways, and additional novel transcripts of both coding and non-coding genes. Tr14 treatment modulated >100 transcripts related to key wound repair pathways, such as response to wounding, wound contraction, and cytokine response. The results provide the most precise and comprehensive characterization to date of the transcriptome's response to skin damage, repair, and multicomponent natural product therapy. By understanding the wound repair process, and the effects of natural products, it should be possible to intervene more effectively in diseases involving aberrant repair.

## Introduction

The cellular and physiological events that comprise the wound healing process remain paramount in the efforts to understand the biological mechanisms of chronic disease. Major diseases, such as atherosclerosis, are understood in the context of aberrant wound repair (Ross, 1993). Even in cancer, the importance of the inflammation system and wound healing has led authors to describe tumors as “wounds that do not heal” (Dvorak, 1986).

Recent advances in systems biology have catalyzed a broad transformation in the conceptual map of inflammation, in particular, the importance of its temporal and spatial evolution. For example, the discovery of the resolvins and their actions within the inflammation system have established the process of “resolution” as a defining event between “acute” and “chronic” inflammation, with the latter often associated with disease progression and poor prognosis (Levy, 2010). Simultaneous with inflammation resolution, wound healing triggers the onset of a coordinated program of regeneration. This program involves migration of resident fibroblasts and epithelial cells, recruitment of adult stem cells and progenitors to the site, followed by differentiation, extracellular matrix production, and tissue remodeling.

Wound repair is a carefully orchestrated response that integrates many of the aspects of embryonic development: coordinated differentiation of progenitor cells, synchronous migration of repair cells, and temporal patterns of cell division, matrix production, and remodeling (Larson et al., 2010). Early inflammation is effectively coupled to subsequent tissue regeneration, but may also contribute to persistent scarring at the site (Eming et al., 2007). While conventional approaches to tissue injury focus largely on suppressing any type of inflammation, via non-steroidal anti-inflammatory drugs (NSAIDs) and glucocorticoids, there is increasing attention toward utilizing multitarget therapies to reduce hyperactivity of the innate immune system (Hwang et al., 2013). Recently, we have proposed a general framework for understanding the scientific basis of regulatory pathways in human disease, termed “Bioregulatory Systems Medicine” (BrSM). BrSM provides a general model for how underlying dysregulations could be corrected using multicomponent products, such as Traumeel (Tr14) (Goldman et al., 2015). Tr14 is a natural product containing 14 plant and mineral ingredients. The exact mechanisms of action are not known, but a number of studies suggest anti-inflammatory (Porozov et al., 2004; Birnesser and Stolt, 2007) and antioxidative properties (Baldwin and Bell, 2007; Zilinskas et al., 2011). Tr14 has shown effects on cellular and cytokine levels in randomized, double-blind controlled trials (RCTs) of exercise-induced muscle trauma (Pilat et al., 2015; Muders et al., 2016, 2017), and demonstrated pain relief in acute ankle sprains (Gonzalez de Vega et al., 2013), with substantial evidence indicating a beneficial role in inflammation and wound repair (Wolfarth et al., 2013).

The present study employed advanced, single-molecule sequencing (SMS) of RNA (RNA-seq) to create a high resolution map of the mouse transcriptome during wound healing, and then uses this map to define changes resulting from therapeutic intervention with Tr14. Transcript profiling by RNA-seq provides a state-of-the-art and relatively bias-free view of the transcriptome that can be used to identify promising networks for evaluating drug actions and biological targets.

## Materials and methods

### Wound healing model

The ICR strain of mice in the age range of 4–6 weeks, ~20 g each, was used for the wound healing studies, under protocols approved by Nantong University Animal Care Committee (PR China). Under sedation (ketamine 100 mg/kg, xylazine 10 mg/kg IP), the mouse dorsal/scapular region was shaved and then a 1 cm^2^ area was abraded with a rotary abrasive tool. Full thickness skin tissue was recovered by sacrifice at specific times: 0, 12, 24, 36, 72, 96, 120, and 192 h after injury. Each time point of each treatment group utilized 7 mice, for a total analysis of 224 mice. Tissue was stored in RNAlater at −80°C until RNA was isolated.

### Treatment conditions

The overall study design is presented in Supplementary Table 1, describing the time points, treatment groups, and individual samples sequenced in the study. Tr14 (Tr14-I group, 9.5 mg/ml) or the saline vehicle (S group) were introduced as 6 subcutaneous injections for a total of 0.1 ml in the region around the wound using a 23G needle placed beneath the abraded area. Other mice received the injections with the addition of twice daily topical Tr14 ointment (0.1 ml of 34 mg/ml) to the wound (Tr14-IO group) or the drug-free ointments control (U group). The injectable solution (bottled in glass ampoules, hydrolytic class I) and ointment Tr14 were manufactured by Biologische Heilmittel Heel GmbH, Germany, according to GMP standards. The study medication was packaged, shipped and labeled by Biologische Heilmittel Heel GmbH, Germany. The full description of ingredients and package labeling is available at the fda.gov website (United States Food and Drug Administration, 2015, 2017).

### RNA isolation for RNA-Seq

Total nucleic acids, enriched for RNA, from the RNAlater-preserved wounded mouse skin was extracted using TRIzol (Invitrogen) using the manufacturer's protocol. The sample was homogenized using rotationally shearing blades (Tissue Tearer) in a 10x volume of TRIzol. The quality and the quantity of the resulting RNA was then measured using absorbance at 260/280 nm on a NanoDrop spectrophotometer (Thermo Scientific) (Chomczynski and Mackey, 1995). Total nucleic acids were DNase-treated (TurboDNAse, 1 ul, 30 min, 37°C) and then depleted of ribosomal RNA using the RiboZero rRNA removal reagents (Epicentre). The quality of RNA was then re-assessed using an Agilent 2100 Bioanalyzer RNA integrity number (RIN > 7) and the sample was quantified using a Nanodrop with a quality threshold of A260/280 > 1.8.

### Transcriptome sequencing (RNA-Seq)

The SMS methodology for transcriptome quantification is described by Lipson et al. (2009), and shown graphically in Supplementary Figure 1. Ribosomal depleted RNA (200 ng) was converted to cDNA using the Superscript III cDNA synthesis kit (Invitrogen) and random hexamer primers, according to the manufacturer's procedure. The RNA remaining in the sample was then degraded by the addition of 1 Unit RNase H and 1 Unit RNase I_*f*_ (New England Biolabs). The resulting cDNA was then purified twice on Performa Gel Filtration Columns (EdgeBio). The concentration of the resulting cDNA was then quantified using a Nanodrop, as previously described (Lipson et al., 2009). To capture the cDNA on the sequencing plates, 3′ poly-A tails were added to the cDNA (100 ng) using terminal transferase. The captured cDNA molecules were directly sequenced by fluorescent nucleotides on the Heliscope single-molecule sequencer (SMS, SeqLL, Inc).

### RNA-Seq data analysis

Each sequencing channel produces an average of 42 million reads per channel, or 43 million reads per sample after double sequencing of channels with low coverage (Supplementary Table 5E). Bioinformatics processing of the data was done using Helisphere 1.2.740 package as previously described (St. Laurent et al., 2013a). Reads were filtered using filterSMS utility with default parameters to remove low complexity sequences, such as poly-A, and short reads under 25 bp. Remaining reads (21 million on average per sample) were then mapped to the mm9 genome (UCSC) combined with ribosomal RNA sequences from NCBI (Accessions: NR_003278.1, J01871.1, NR_003279.1) using indexDPgenomic aligner (Lipson et al., 2009; St. Laurent et al., 2013a). Only reads with single best alignment at or above the minimal alignment score of 4.3 were considered. Up to 30% of reads map to Ribosomal RNA sequences and are intentionally filtered out, thereby preventing mis-mapping of those reads to the rest of genome. On average, 7.1 million reads per sample, or 33% of filtered reads satisfy mapping criteria—having unique best mapping position on chr1-19, X, Y, M (Supplementary Table 5E). Such mapped reads are called Informative Reads in downstream analysis. Considering the short read length, requirement of unique alignment, and platform properties, the proportion of Informative Reads relative to filtered reads is within the expected range. Informative Reads were mapped to transcripts based on the knownGene.txt file (mm9 version of the mouse genome) in UCSC Genes database (Kent et al., 2002). The number of Informative Reads overlapping exonic intervals in genome in each transcript is calculated, and converted to units of RPKM (reads per thousand (K) nucleotides length of spliced transcript, per million reads captured per sample) using custom Perl script. Previous studies confirm that quantitative expression levels generated by this process are able to detect changes of < 2-fold, even at quite low absolute RNA abundance (St. Laurent et al., 2013a).

### Differentially expressed genes (DEG)

Fold-change (log_2_ scaled) and *p*-value (probability to obtain the fold change by chance) for each transcript were compared between groups of samples using Welch's test and edgeR Bioconductor software package (Robinson et al., 2010). The parameters were tuned to make biological properties of selected genes more prominent and minimize the false discovery rate (FDR). For the comparison of Tr14-treated group (Tr14-I or Tr14-IO) vs. placebo saline treated (S), edgeR unadjusted *p*-value was required to be below 0.0005. The common dispersion, trended dispersion and tagwise dispersion of raw (not RPKM normalized) expression counts were considered when fitting parameters of overdispersed Poisson model by edgeR software. For the timecourse U vs. 0 h U comparison, the Welch's test was used and unadjusted *p*-value cutoff was required to be below 0.001. Results of comparison of U vs. U at 0 h groups with edgeR were not used because large differences between wounded and unwounded tissues made the results of the highly sensitive edgeR tool too broad and unfocused.

For differentially expressed genes, the fold change between RPKM values averaged across the samples was required to be at least 1.41 (or 0.5 in log2 scale). Maximum 2000 differentially expressed genes, defined in this way with the lowest *p*-value, were selected for the subsequent analysis. This threshold was applied to make gene ontology enrichment analysis comparable between different groups, because GO analysis heavily depends on the number of input genes. In fact, at every time point of U vs. 0 h U timecourse the 2000 cutoff was substantially applied, which kept the FDR below 0.025.

### Systems biology data analysis

To understand correlations and patterns among different elements of the transcriptome, differentially expressed RNAs are mapped to their respective biological pathways and Gene Ontology (GO) categories (Harris et al., 2004) using DAVID software (Huang da et al., 2009). Changes enriched in individual pathways or ontologies were tested for their statistical significance as measured by a number of hits greater than expected by chance, with a *p* < 0.1, calculated using a hypergeometric model. GOTERM_BP_DIRECT table from “Functional annotation” output of DAVID was taken as the result. Specific pathways were analyzed and studied with Pathway Studio (Nikitin et al., 2003) and ExPlain software (Kel et al., 2006).

## Results

### Mouse wound healing response to partial-thickness abrasion

The histological characterization of the partial-thickness abrasion model employed here has been well described by others (Gupta et al., 2014). The murine wound repair process has recently been elegantly detailed using intravital imaging of punch wounds to provide an excellent temporal description of cellular migration, proliferation, and differentiation (Park et al., 2017). Consistent with the very efficient cutaneous wound repair processes in the mouse, there were no striking changes in the gross morphological repair process induced by Tr14, and so the effects observed on gene expression are likely not due to major cellular changes in the harvested wound regions.

### Gene expression profiling of the wound healing response

Although over 108,000 references relating to wound healing exist in the PubMed database, relatively few provide data on gene expression. Currently, the only published RNA-seq dataset profiling a wound healing time course covers a model of bone healing in sheep (Jager et al., 2011). To describe the changes in the mouse skin transcriptome during normal wound healing, an analysis of gene expression changes in the control time courses was conducted using a combined fold-change/*p*-value method at each point after injury. Collectively, the RNA-seq analysis represents over 3.9 billion sequence reads, which is currently among the largest RNA-seq datasets for skin wound healing in existence, comparing favorably in scope with a recent RNA-seq analysis of repair after cryo-induced muscle injury (Aguilar et al., 2015).

Supplementary Figures 2A–G present Tagwise Dispersion Plots showing the distribution of genes by expression level (X-axis), and fold change (Y-axis) for the 12 h time-point through the 192 h time points of the control wounds (U) relative to Time 0. Genes marked in red represent the subset that has statistically significant fold change measurements.

Supplementary Table 2 lists the top 100 genes up or down-regulated at each wound healing time point, their relative expression levels as measured by normalized read counts, and *p*-values. The list highlights hundreds of genes from the major mammalian pathways critical to wound healing, such as cytokines, modulators of tissue organization, growth factors, and other genes active in the wound healing process.

### Wound healing pathway analysis

The above results demonstrate hundreds of genes up- and down-regulated during the time course of wound healing. In order to achieve a better systems-level understanding of how the wound healing system engages transcripts at each phase of repair, Gene Ontology (GO) analysis was employed. GO facilitates a formalized, consistent vocabulary for understanding the functional categories of many genes in a system. The engagement of the Response to Wounding GO is demonstrated by the modulation of 53 genes in this category at 12 h post-injury, which is far more than would be expected randomly (14 genes expected, *p* < 10^−46^), see Supplementary Figure 3 for details.

**Figure 1:**
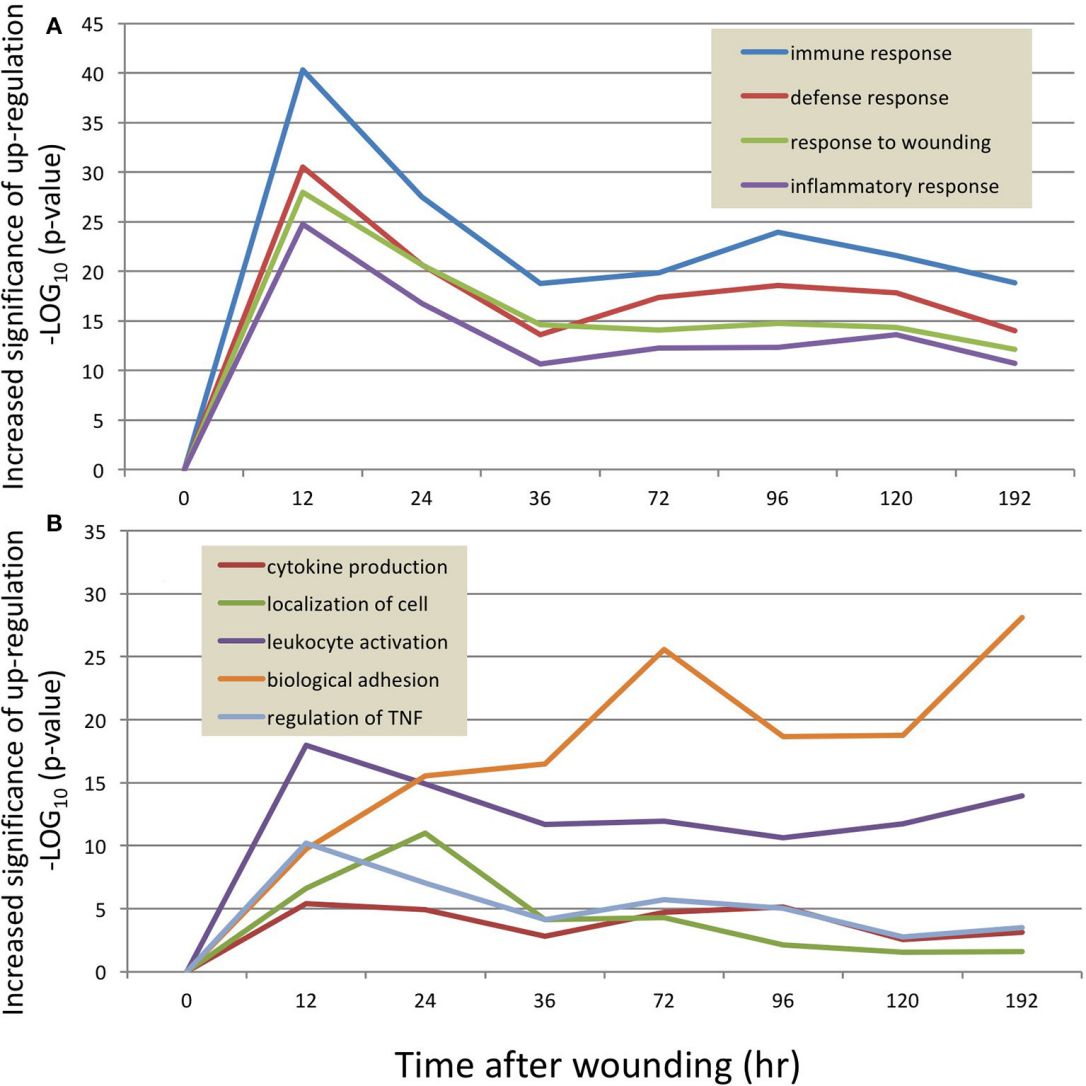
The temporal response of the transcriptome to wounding of the mouse dermis. RNA sequencing results were analyzed at each time point (X axis) to identify transcripts which differed significantly from the 0 h uninjured control sample. The resulting transcripts were then categorized into pre-curated ontologies, and the ontologies with the greatest activity are plotted as a function of the probability that the ontology is activated disproportionately to a random sample of transcripts (Y-axis, *p*-value, log_10_scale). Because an ontology may contain ~200 transcripts, at any time point there might be transcripts that are increased or decreased in magnitude relative to the control time point. **(A)** shows 4 ontologies with the highest statistical probability of being increased at the 12 h time point, whereby the “immune response” ontology (blue line) is over-represented at a probability of ~10^−40^ compared to a random set of transcripts. **(B)** depicts 5 ontologies that are engaged in the 24–72 h time frame.

Figure 1 organizes these groups of transcripts according to early, middle and late responding changes in placebo-treated wounds (U). As expected, the 12-h time-point demonstrates an intense signal for wound healing genes (Figure 1A). At the 12 h time point, there is very striking engagement of the Defense Response, Immune Response, Response to Wounding, and the Inflammatory Response, as demonstrated by strong over-representation of these transcripts [-log(p-value) on Y-Axis, i.e., 40 is 1^*^10^−40^ probability that the Immune Response transcripts could have been affected by chance alone.] Figure 1B demonstrates that certain processes are sustained into the later time frames with at least 80% of peak expression at 24 h or later. Interestingly, this cluster of GO categories with sustained engagement includes Localization of Cell, Leukocyte Activation, Cytokine Production, Biological Adhesion, and TNF Production. Within this 8-day period, the majority of inflammatory and wound repair processes have engaged, and then subsided, giving way to transcriptional programs designed to restore tissue integrity, such as extracellular matrix synthesis and wound contraction.

**Figure 2:**
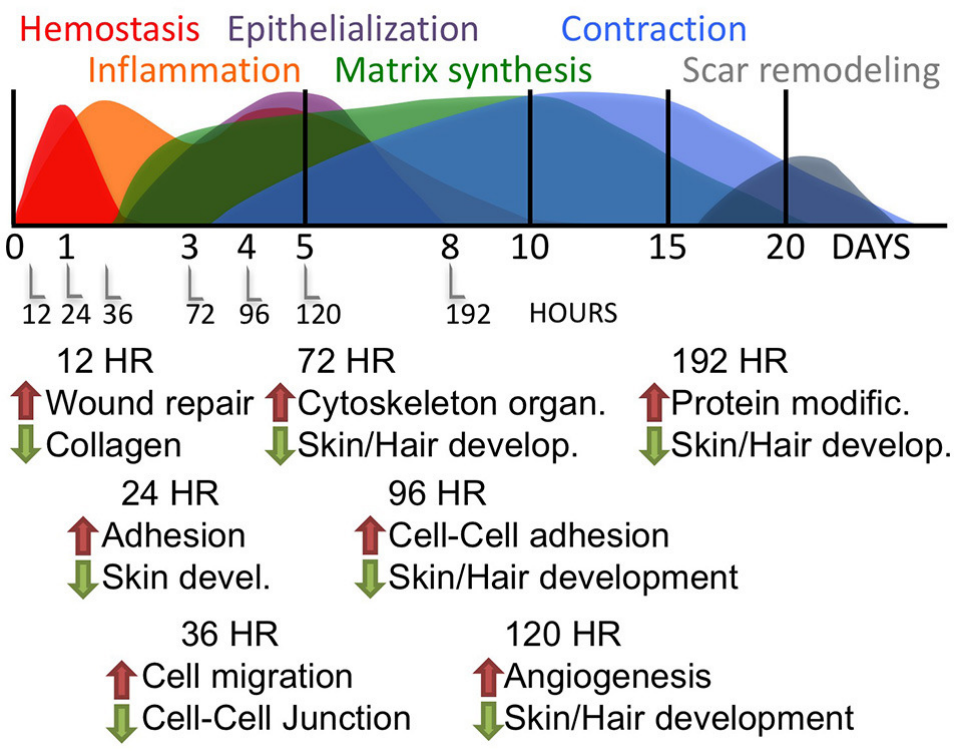
Time-course of RNA response to skin wounds. **Upper panel:** A graphic summary of the major pathways engaged during mammalian wound repair as adapted from Seifert et al. (2012). **Lower panel:** The major gene ontologies of the transcripts regulated by wounding of the mouse skin, as detailed in Table 1.

Collectively, these data demonstrate a detailed and high-resolution picture of the transcriptional landscape of murine wound healing. Table 1 lists the principal GO categories regulated (up and down) at each post-wound time point. Figure 2 summarizes these transcriptome changes and overlays the present results onto previously published descriptions of the progression of cutaneous wound repair, as recently reviewed (Seifert et al., 2012).

**Table 1:** Top Gene Ontologies changed at each time point.

	**Top 8 GO UP regulated**	**Top 8 GO DOWN regulated**
	GO Term^*^	GO Term^*^
12 HR	immune response	regulation of system process
	defense response	membrane depolarization
	response to wounding	regulation of muscle contraction
	cell activation	regulation of heart contraction
	inflammatory response	regulation of striated muscle contraction
	positive regulation of response to stimulus	regulation of interleukin-10 production
	cytokine-mediated signaling pathway	oxygen transport
	chemotaxis	regulation of interleukin-4 production
24 HR	cell adhesion	melanin biosynthetic process
	biological adhesion	melanin metabolic process
	angiogenesis	nucleosome assembly
	vasculature development	chromatin assembly
	blood vessel development	chromatin assembly or disassembly
	blood vessel morphogenesis	nucleosome organization
	actin filament-based process	protein-DNA complex assembly
	regulation of cell proliferation	DNA packaging
36 HR	glycoprotein metabolic process	ectoderm development
	vesicle-mediated transport	epidermis development
	blood vessel morphogenesis	keratinocyte differentiation
	blood vessel development	epithelial cell differentiation
	vasculature development	epidermal cell differentiation
	actin filament-based process	epithelium development
	biopolymer glycosylation	keratinization
	glycosylation	transition metal ion transport
72 HR	cell adhesion	ectoderm development
	biological adhesion	epidermis development
	actin filament-based process	epithelial cell differentiation
	actin cytoskeleton organization	keratinocyte differentiation
	cytoskeleton organization	epidermal cell differentiation
	cell-cell adhesion	keratinization
	homophilic cell adhesion	transition metal ion transport
	membrane invagination	molting cycle
96 HR	cell adhesion	ectoderm development
	biological adhesion	epidermis development
	phosphate metabolic process	keratinocyte differentiation
	phosphorus metabolic process	epidermal cell differentiation
	actin filament-based process	keratinization
	protein amino acid phosphorylation	epithelial cell differentiation
	homophilic cell adhesion	hair cycle
	actin cytoskeleton organization	molting cycle
120 HR	blood vessel development	ectoderm development
	vasculature development	epidermis development
	protein amino acid phosphorylation	epithelial cell differentiation
	positive regulation of molecular function	epithelium development
	phosphate metabolic process	mesoderm development
	phosphorus metabolic process	keratinocyte differentiation
	enzyme linked receptor protein signaling pathway	epidermal cell differentiation
	angiogenesis	mesoderm formation
192 HR	vesicle-mediated transport	ectoderm development
	phosphorus metabolic process	epidermis development
	phosphate metabolic process	keratinocyte differentiation
	protein amino acid phosphorylation	epithelial cell differentiation
	actin filament-based process	epidermal cell differentiation
	cell adhesion	translational initiation
	biological adhesion	mesoderm development
	homophilic cell adhesion	keratinization

* *Ontologies presented in order of lowest to highest p-values of Top 8*.

### The effects of Tr14 on gene expression during wound repair

The RNA-seq data indicates that Tr14 treatment produced biologically significant and consistent changes in hundreds of genes involved in wound healing pathways. Of note, the technical characteristics of the SeqLL/Helicos SMS technology have proven accuracy and reproducibility even at low (1.5X) to moderate (2X) changes in gene expression (St. Laurent et al., 2013a,b). Published comparisons of tSMS RNA-seq to RT-PCR has observed correlations of 0.85–0.90 between these methods (St. Laurent et al., 2013b, 2016). Regardless of the mode of delivery, Tr14 treatment resulted in hundreds of differentially regulated genes compared to the Saline control group. For example, at 96 h, the Tr14-IO group resulted in 21 differentially expressed genes from the Response to Wounding GO category, while the Tr14-I group resulted in 31 differentially expressed genes. Notably, these two sets overlapped by 13 genes, over 50% of the maximum potential overlap (*p* < 2.1 × 10^−10^, Fisher's exact test, Supplementary Figure 4), demonstrating that 2 different forms of Tr14 treatment produced many similar transcript changes, while also emphasizing that topical and injectable delivery produced somewhat different responses. Similar concurrence between the two forms of delivery was observed in the Epithelial Cell Differentiation GO category (Supplementary Figure 5).

### Expression changes in key wound healing pathways resulting from Tr14 treatment

**Figure 3:**
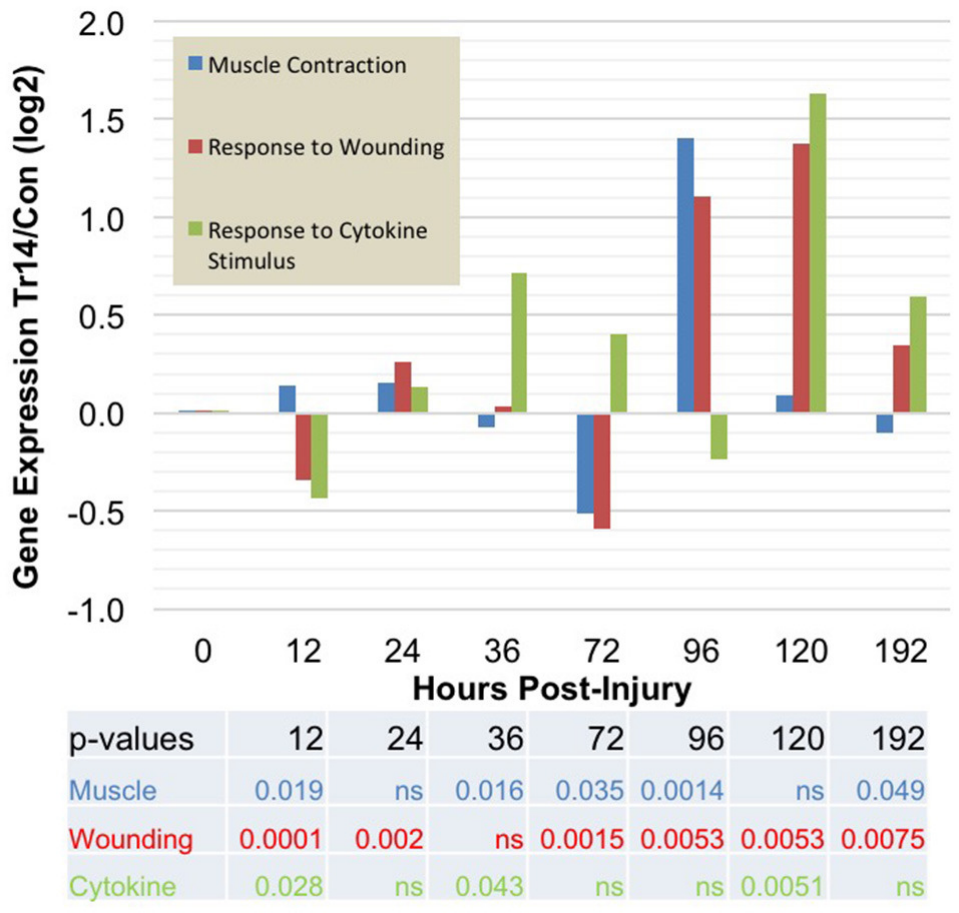
The effect of Tr14 on gene expression patterns after wounding. The RNA sequencing results were analyzed to identify transcripts that were differentially expressed between placebo-treated controls and Tr14-treated wounds, at each time point after injury (X axis). The effect of Tr14 on a given ontology is shown as an aggregate gene expression score of the differentially expressed transcripts in that GO group, expressed as the log2 ratio of the Tr14 vs. control levels, (Y axis, log2 ratio Tr14/Con). For the Muscle Contraction GO, a total of 25 transcripts were affected covering 17 unique, non-redundant genes. Tr14 affected 87 transcripts (51 genes) in the Response to Wounding GO, and 9 transcripts (6 genes) in the Response to Cytokine Stimulus GO.

Differentially regulated genes, either increased or decreased by Tr14-IO compared to control are summarized by their principal Gene Ontologies in Figure 3. A relatively large number of gene expression changes occur in the time-course as a result of Tr14 treatment. There was a noticeable effect of Tr14 on the Response to Wounding and Response to Cytokines Gene Ontologies, peaking by about 96–120 h post-injury and treatment (Figure 3). Several categories exhibited secondary peaks at 96–120 h, notably, Anti-apoptosis and Cell Differentiation (not shown). Notable was a strong engagement of the Muscle Contraction ontology at 96 h, which involves transcripts that are related to wound contraction (Figure 3). As shown later in Figure 6, about 25% of the transcripts in the muscle contraction pathway were affected at 96 h.

### Selected wound repair transcripts affected by Tr14

Using the time-course as an additional filter, transcripts with changes in multiple time-points were identified because they are more likely to be important factors in the Tr14-mediated physiological network. In fact, the majority of these genes fall into categories relevant to wound healing and provide potential targets for further study of the molecular mechanisms of Tr14 action.

**Figure 4:**
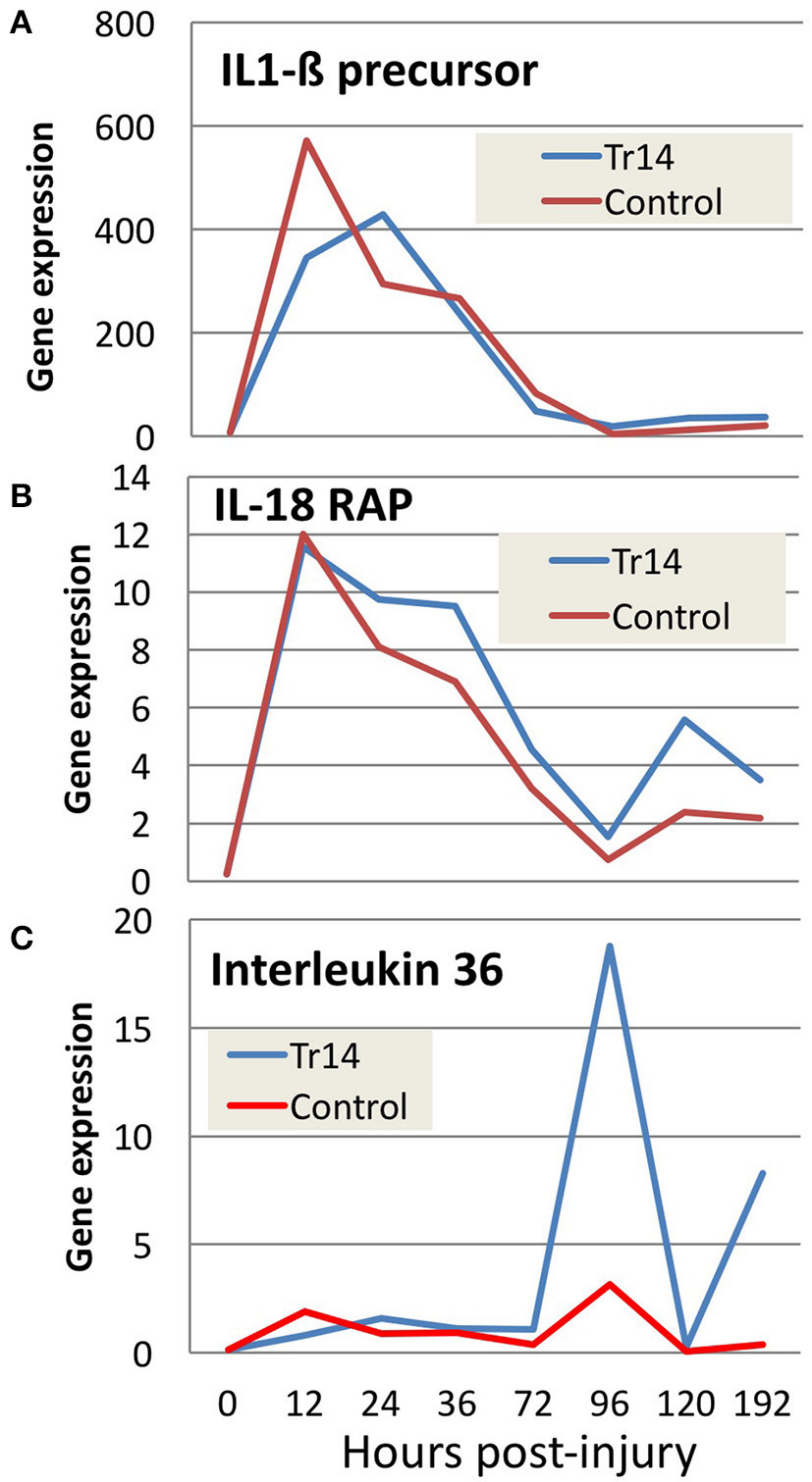
The effect of Tr14 on select transcripts in the interleukin pathway. RNA sequencing data was analyzed to identify transcripts modulated by Tr14 treatment. Significant increases in three of the transcripts **(A–C)** in the interleukin pathway are shown as gene expression (RPKM, Y axis) as a function of time in hours (X axis).

For example, Tr14 treatment regulates a number of genes in the Interleukin family, which play key roles in intercellular communication between lymphocytes and a variety of other immune cell types. Figures 4A–C shows the relative expression of Interleukin family members in Tr14-treated animals compared to controls. Figure 4A shows that Tr14 treatment delays and attenuates by 30–40% the strong increase (~600-fold) in IL1ß mRNA expression in the 12–24 h period after wounding. Produced by monocytes and macrophages, IL1ß is an important mediator of the inflammatory response (Mirza and Koh, 2015). IL1ß induces cyclooxygenase-2 (PTGS2/COX2), which is a key enzyme in the production of thromboxanes, leukotrienes, and HETEs, which directly contributes to inflammatory pain and hypersensitivity (Burke and Collier, 2011). The reduction of IL1ß mRNA levels is consistent with Tr14's known action to reduce the pain of inflammation in other indications (Schneider, 2011; Gonzalez de Vega et al., 2013). Other plant-derived therapeutics, such as resveratrol, are also known to attenuate IL1ß levels after burn injuries (Tao et al., 2015).

Figure 4B shows a comparison of mRNA expression levels of IL18 receptor accessory protein (IL18RAP) in Tr14-treated vs. controls. The initial wound causes an almost identical increase of ~12-fold above baseline IL18RAP levels in treated and untreated wounds. However, at each of the following 6 time points, the Tr14-treated group shows a moderate, but reproducible increase in expression levels. IL18RAP has been shown to regulate interferon-γ production in peripheral blood monocytes (Myhr et al., 2013), and so this increase might reflect altered monocytic infiltration.

Figure 4C demonstrates the effect of Tr14 on IL36 expression in the mouse wound. Several studies implicate IL36 in inflammatory skin conditions in mice (Blumberg et al., 2007), and in human psoriasis (Towne et al., 2011). Interestingly, the fold-change of IL36 in treated animals increases to as much as 20-fold higher in Tr14-treated wounds at 96 h post-injury. Injury caused as much as 10-fold inductions of IL-6, IL-8, and IL-10 mRNA, with a relatively mild effect of Traumeel over time (Supplementary Figure 6).

**Figure 5:**
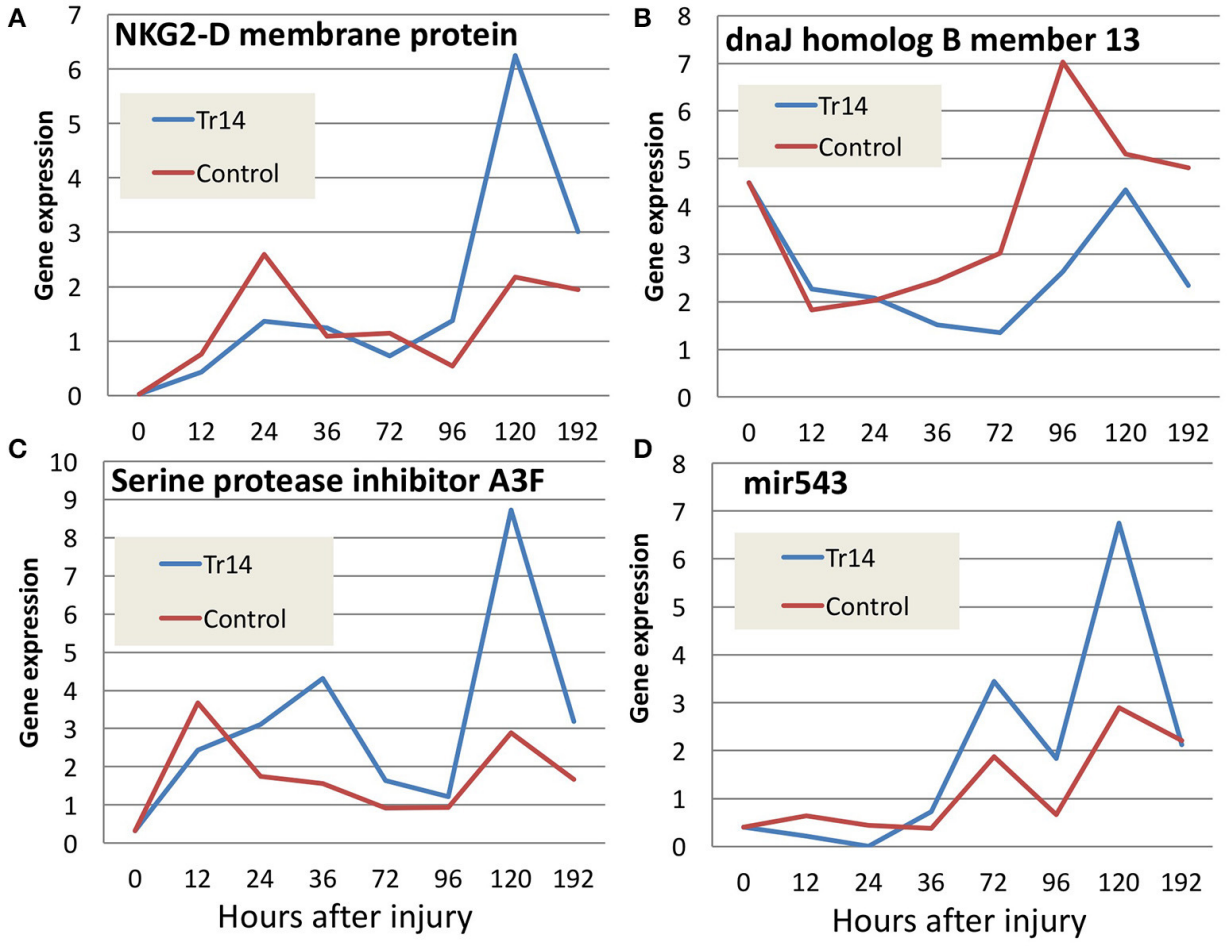
Comparison of gene expression for selected transcripts **(A–D)** in Tr14 treated animals vs. saline controls. The X axis reports the time after injury to the mouse skin, when the wounded region was harvested for RNA and quantitation by RNA-seq. The Y axis reflects the average normalized gene expression for the transcripts specified in each panel. Red lines indicate expression levels in control mice and blue lines indicate levels in Tr14-treated wounds (IO Group).

A variety of other genes important in wound healing display changes in expression along the time course as a result of Tr14 treatment and are listed in Supplementary Table 3. The false discovery rate for this list is 0.159, 0.557, 0.238, 0.066, 0.024, 0.019 and 0.026 for 12, 24, 36, 72, 96, 120, and 192 h time points, respectively. Some examples include cell stress and damage markers (NKG2-D, DNAJ B), extracellular matrix-regulating genes (serine protease inhibitor A3F), and miRNAs (mir543) (Figures 5A–D). NKG2D (KLRK1), for instance, is a natural killer cell receptor which has an extensively published connection with the innate immune response system's ability to detect stress and senescent cells (Sagiv et al., 2016), and thus, along with its ligands, is considered a therapeutic target for immune diseases (Gonzalez et al., 2008). DNAJB13 is a member of the heat shock protein (HSP) family, and has recently been associated with defects in cell motility (El Khouri et al., 2016). Serine protease inhibitor A3 is likely involved in controlling elastase activity and members of this family have been implicated in maintaining barrier functions (Hu et al., 2013). Micro RNA 543 (mir543) has been associated with mesenchymal stem cell differentiation, and thus may be related to changes in the cellular differentiation pathways (Xu et al., 2017).

### Principal gene ontology categories affected by Tr14 treatment

The Gene Ontology mapping of the RNA-seq dataset provides a means of identifying patterns of gene expression during wound repair. A complete list of Gene Ontology categories significantly affected by Tr14-IO treatment at each time-point is included in the Supplementary Table 4. One important group of genes regulated by Tr14 treatment includes modulators of tissue organization and pattern development. For instance, at 12 h post-injury, a cluster of key modulators of the developmental process are affected by Tr14 including Desmoglein 4 (Dsg4), peroxisome proliferator activated receptor-γ (Pparg), and the keratins family (Krt). Among the most intriguing Gene Ontology categories affected by Tr14 in this study were the categories of Muscle Contraction, Response to Wounding, and Response to Cytokine Stimulus as shown in Figure 3, Tr14 upregulates a relatively large number of these genes, especially during the 96–192 h time frame.

The data reveals a similar, but inverse effect of Tr14 on the Epithelial Cell Differentiation gene ontology category at later time points. Supplementary Figure 5 details only genes in the Epithelial Cell Differentiation GO which are up and down regulated in either of the two Tr14 treatment groups. At two time-points, there is a >50% overlap in these differentially expressed genes between the two treatment groups, and with a highly significant *p*-value. Overall, the broad changes in cellular differentiation genes in Tr14 treated groups suggests an enhanced regulation of the differentiated state in the microenvironments of Tr14-treated wounds. This effect would increase the repair capacity in the area of the wound, and could explain another fundamental aspect of Tr14's therapeutic effect.

### Network analysis provides hypotheses for Tr14 mode of action

**Figure 6:**
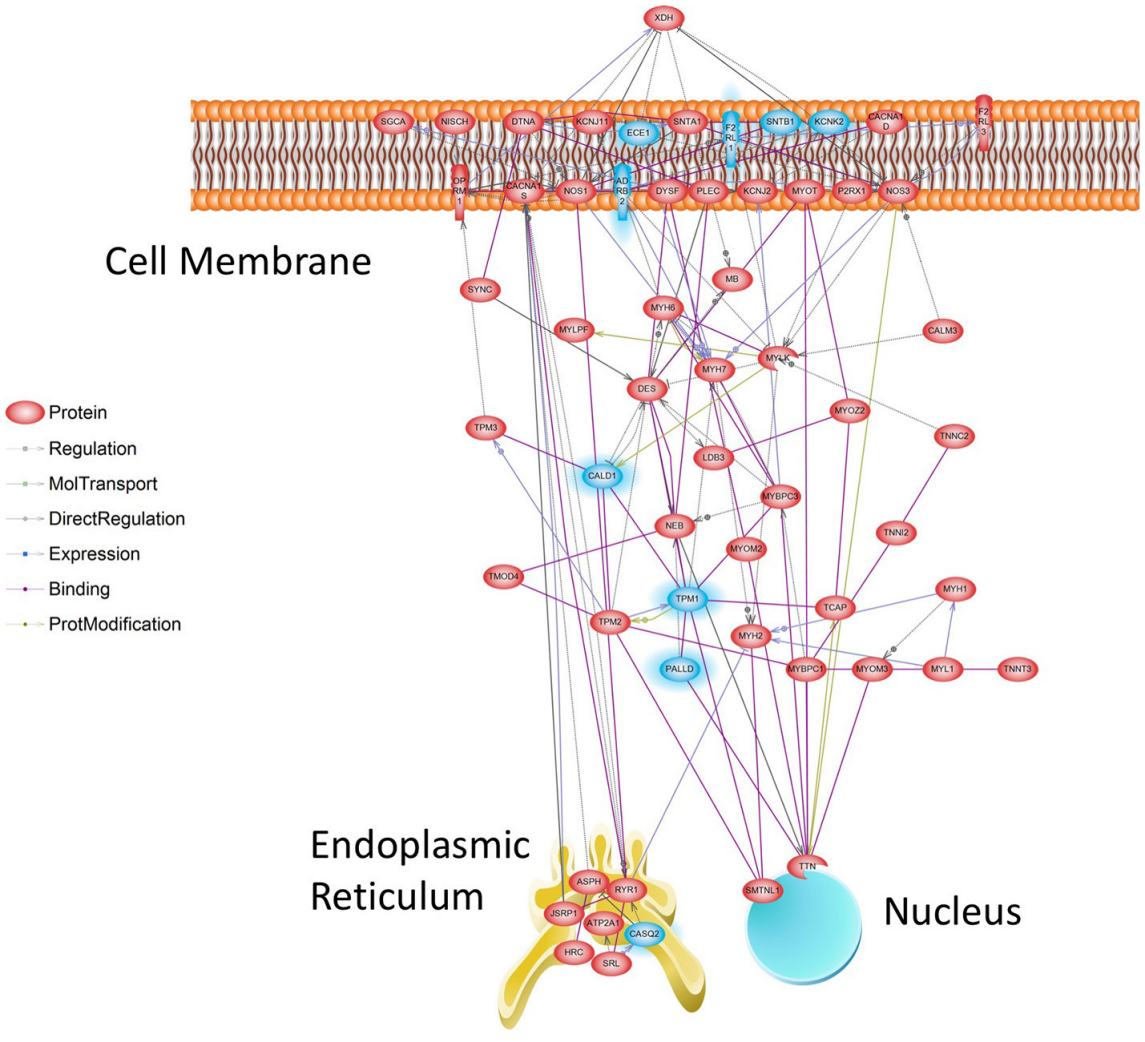
Muscle Contraction Gene Network at 96 h. Transcripts affected by Tr14 treatment were analyzed for their relevance to a series of pre-established biological pathways as described in Methods (Systems Biology). A greater than expected number of transcripts belonged to the Muscle Contraction pathway, which likely reflects wound contraction processes. In this pathway, reconstructed from the literature by Pathway Studio software (Nikitin et al., 2003), individual transcripts described by their standard gene symbol are connected by lines. Lines may connect genes if one gene product is known to induce the expression of another, if products interact directly, or if one product is a substrate for the other. Transcripts that are increased by Tr14 are highlighted in RED, while transcripts that decreased are shown in BLUE.

While GO Categories represent broad collections of genes grouped by biological functions, effectively a “Parts List,” biological networks equate to detailed “Wiring Diagrams” that include regulatory circuits, feedback loops, and control elements. The Muscle Contraction ontology was selected for further analysis because it was strongly affected by Tr14, and it is a well-characterized network of genes with a common, well-defined function. To analyze Muscle Contraction in the form of a network, we developed a methodology to filter network nodes and edges collected from the literature by selecting only differentially expressed nodes for the final network representation, which would emphasize nodes that are susceptible to regulation. At 96 h, 65 of the 249 transcripts in this pathway were affected by Tr14 treatment, which is vastly larger than would be expected by chance alone (*p* = 7.8 × 10^−29^). The resulting Muscle Contraction network, depicted at the 96 h time point in Figure 6, shows a set of transcripts that are associated with wound contraction and regulated upward (red) or downward (blue). Thus, using this preconstructed pathway (Nikitin et al., 2003) as a molecular map, it could be concluded that transcripts in the wound contraction pathway are affected by Tr14 treatment, possibly contributing to a therapeutic effect of Tr14.

## Discussion

The physiological process of wound healing provides an integrated model for injury, inflammation, and repair. As the most extensively annotated non-human mammalian genome, the mouse provides many advantages for genomic studies, especially in the extensive ontologies and pathways available for a Systems Biology analysis. The systems biology of wound repair involves many of the processes which can become dysregulated in chronic diseases. Tissues affected by chronic diseases display physiological processes similar to those involved in aberrant wound healing, including the tendency toward fibrosis and lack of effective healing.

This study of the transcriptome during wound repair, and Tr14 treatment, explores for the first time the changes in genomic expression patterns resulting from a multicomponent, multitarget medication in the context of a complex physiological process critical to disease treatment. The study includes a database of almost 4 billion sequence reads covering 224 animals, 4 conditions, and 8 time-points. With respect to read depth, time points, and biological replicates, it is among the largest RNA-seq studies conducted in wound healing completed to date, and the first involving a multicomponent therapy.

The results indicate that a number of important gene expression networks are involved in wound healing. These changes arguably reflect two general types of changes: (1) changes in the gene expression of the cells in injured tissue, and (2) the influx of new cell types into the wounded area. The influx of new cell types in response to skin damage, such as macrophages and lymphocytes, likely influences the expression of a number of regulated genes. Likewise, paracrine signaling, cell death, and reprogramming of the cellular state also generate coordinated changes in gene expression within resident cells of the wound tissue.

Tr14 treatment results in extensive gene expression changes during wound healing, including well-known pathways, such as TGF-β, cytokine signaling, inflammation, wound contraction, collagen, and enzymes of the extracellular matrix. Interestingly, both of the Tr14-treated groups revealed broad and statistically significant changes in three Gene Ontology groups of great importance to wound healing: Response to Wounding, Response to Cytokines, and Muscle/Wound Contraction. These signals may indicate effects upon resident fibroblasts and infiltrating immune cells, which could easily have been overlooked in simpler experimental models. This result agrees with clinical evidence that Tr14 therapy improves a variety of musculoskeletal injuries, including the associated pain and swelling (Schneider, 2011).

The evidence of broad regulation of genes in these three Gene Ontology categories has interesting implications for the mode of action of Tr14. The patterns of expression changes shared by Tr14-IO and Tr14-I treatments suggest two intriguing hypotheses for Tr14 action in diseased tissue. The consistent regulation of many genes related to cell differentiation suggests an effect on the cellular state in the microenvironment of the wound. The net effect could promote a more effective transition to a less differentiated, more pluripotent state among cells in the microenvironment. At the same time, changes in expression of “Cell motility” genes suggest that Tr14 facilitates cell migration and tissue organization during the wound healing process. Increased support of pluripotency could be synergistic with the facilitation of cell mobility to improve tissue regeneration in a variety of disease and damage contexts.

Combined, the present studies demonstrate that while Tr14 had no observable effect on morphological repair of mouse skin, it had a clear effect on gene expression in the wound. Mice, like most rodents, have very efficient wound repair mechanisms, and rarely exhibit the types aberrant scarring that can be common in humans (Rittie, 2016). Thus, one should interpret mouse wound healing studies with caution in respect to potential beneficial effects in humans. It is reasonable to speculate that the changes in gene expression observed in this rodent model indicate that this multicomponent, multitarget therapy has perceivable effects on key aspects of wound repair, and should be studied further in human skin disorders and other inflammatory conditions.

## Data accessibility

The raw RPKM data, including each animal at each time point in every group, is submitted for public access in the Supplementary Table 5 folder.

## Author contributions

Conceived and designed the studies: GS, BS, and TM. Performed the experiments: JZ, IT, MT, DJ. Analyzed the RNA-seq data: DS, YV, GS, YV, and TM. Wrote the paper: GS and TM. Commented and edited the manuscript: all authors.

### Conflict of interest statement

As disclosed above, the studies were partially funded by Biologische Heilmittel Heel GmbH, a for-profit company, and the St. Laurent Institute, a non-profit institute.

## Supplementary material

Supplementary Figure 1 The schematic workflow of RNA sequencing using the SeqLL true Single Molecule Sequencing (tSMS) method. Total RNA from RNAlater-preserved skin samples at the wound site was isolated by Trizol, and then depleted of ribosomal RNA (riboRNA) prior to fragmentation for RNA-seq. The fragments are tailed with polyA to allow capture by polyT strands on the sequencing plate. True Single Molecule Sequencing was conducted producing about 42 M total reads, and ~21 M short reads (25–65 nt) per channel, after filtering. The filtered short reads are aligned to the mouse genome and then reads within exons are counted and summed per transcript. The raw read count is adjusted for the length of the transcript and the total number of aligned reads obtained for that sample.

Supplementary Figure 2 **(A–G)** Tagwise dispersion plots of wound-related transcripts in untreated control mice at specified times after injury. RNA-seq data, expressed as RPKM, was analyzed for differential expression between the 0 h, unwounded, and specific time points afterward. The log_2_ RPKM gene expression for each transcript (red or blue circle) is ratioed between sample time points to create differential expression as a fold change (Y axis) vs. the absolute level of expression of the transcript (X axis). Transcripts highlighted in red are the top 2,000 most differentially expressed (DE) between the stated time points.

Supplementary Figure 3 Graphic Representation of the intensity of the Response to Wounding GO category at specific time points of the wound healing response. Details of total transcripts affected (hits), total transcripts in pathway, hits expected by chance, and *p*-value, for the Response to wounding/Wound Healing GO category, at each time point.

Supplementary Figure 4 The effect of Tr14 treatment on transcripts in the Response to Wounding GO. Upregulated transcripts were calculated between Tr14 vs. saline-treated controls at each of the time points specified as described in Materials and Methods except a *p*-value threshold of 0.01 was applied. The upregulated transcripts were analyzed for preferential enrichment of the Response to Wounding GO. The two types of Tr14 treatment are shown in separate columns and the overlapping transcripts with Fisher's exact test *p*-value are counted in the right columns.

Supplementary Figure 5 The effect of Tr14 treatment on transcripts in the Epithelial Cell Differentiation GO. Details are similar to Supplementary Figure 4 except that both up and down-regulated transcripts in the Epithelial Cell Differentiation GO were analyzed.

Supplementary Figure 6 The effect of partial-thickness abrasion and Tr14 treatment on IL6, IL8, and IL10 mRNA levels. The levels of 3 well-characterized interleukins is shown over time after injury of the mouse skin. Control wounds treated with saline (S), are compared to wounds treated with Tr14 as a combination of injections and topical treatment as specified (IO, labeled G here).

Supplementary Table 1 Overall study design, describing the time points, treatment groups, and individual samples sequenced in the study.

Supplementary Table 2 The top 100 differentially expressed in control wounds compared to the unwounded, 0 h time point. RNA-seq data was analyzed to identify transcripts affected by abrasive wounding of the mouse skin at the specified time points. Differentially expressed transcripts were identified by statistical comparison using Welch's test (*P*-value). The fold change of the difference between expression levels is reported as a log_2_ value (Log_2_Fold). The absolute level of expression for each UCSC ID transcript is described for each time point in RPKM. The top 100 transcripts elevated (0 < 12 h) or decreased (0 > 12 h) is listed for each tested time point.

Supplementary Table 3 Differentially expressed transcripts in Tr14-treated wounds compared to control. RNA-seq data was analyzed to identify transcripts modulated by Tr14 during wound repair. The transcripts with the *p* < 0.0005 and a fold-change > 1.41 are shown for each of the time points from 12 to 192 h. For each time point, the RPKM values are shown for each sample and each UCSC ID. The log_2_ fold change and *P*-value by EdgeR are also shown for each transcript.

Supplementary Table 4 Gene ontology (GO) categories for differentially expressed genes affected by Tr14 treatment of wounds over time. RNA-seq data was analyzed to identify transcripts modulated by Tr14-IO during wound repair, as shown in Supplementary Table 3. The transcripts were then organized using DAVID (Huang da et al., 2009) into Gene Ontologies that covered those transcripts at each time point post-injury.

Supplementary Table 5 Raw data for RNA sequencing analysis of mouse wound repair. The raw RPKM data for gene expression for all groups, animals, and time points is reported in the following tables. **(A)** Reports the (U) group of placebo treated animals, **(B)** reports the (S) saline injected control group, **(C)** reports the (I) Traumeel injection group, and **(D)** reports the (IO) Traumeel injection plus the Traumeel ointment group. **(E)** Reports a summary of the sequencing data with respect to total raw reads, filtered reads, Informative Reads and the number of expressed transcripts for each sample in the study, by group.
