# Supplementary figures and images for: Deep Sequencing Transcriptome Analysis of Murine Wound Healing: Effects of a Multicomponent, Multitarget Natural Product Therapy-Tr14
Source: Front Mol Biosci. 2017 Aug 17;4:57. doi: 10.3389/fmolb.2017.00057 (PMC5572416; doi:10.3389/fmolb.2017.00057)

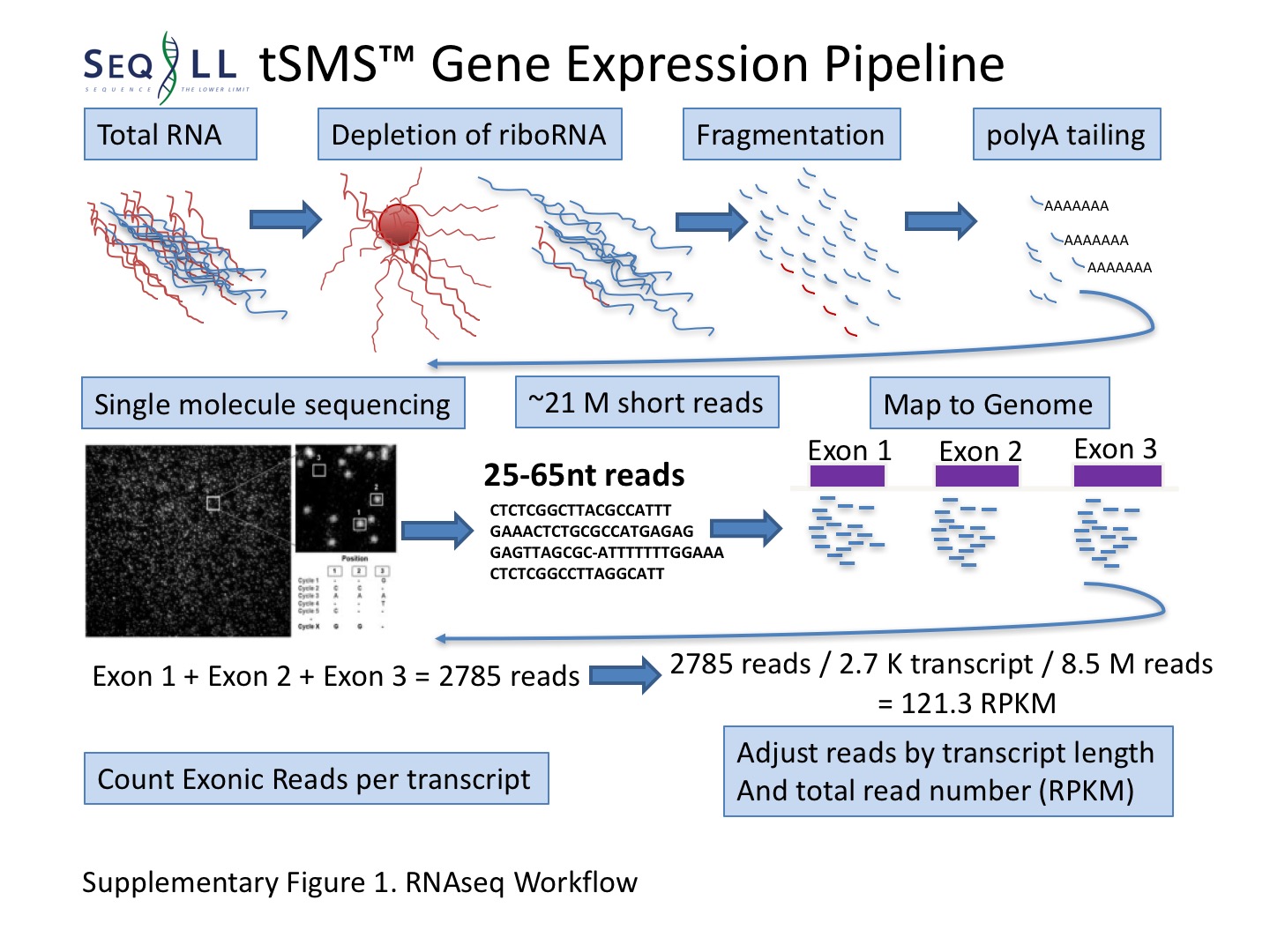

Supplement: Supplementary Figure 1 — The schematic workflow of RNA sequencing using the SeqLL true Single Molecule Sequencing (tSMS) method. Total RNA from RNAlater-preserved skin samples at the wound site was isolated by Trizol, and then depleted of ribosomal RNA (riboRNA) prior to fragmentation for RNA-seq. The fragments are tailed with polyA to allow capture by polyT strands on the sequencing plate. True Single Molecule Sequencing was conducted producing about 42 M total reads, and ~21 M short reads (25–65 nt) per channel, after filtering. The filtered short reads are aligned to the mouse genome and then reads within exons are counted and summed per transcript. The raw read count is adjusted for the length of the transcript and the total number of aligned reads obtained for that sample. [file Image1.JPEG]

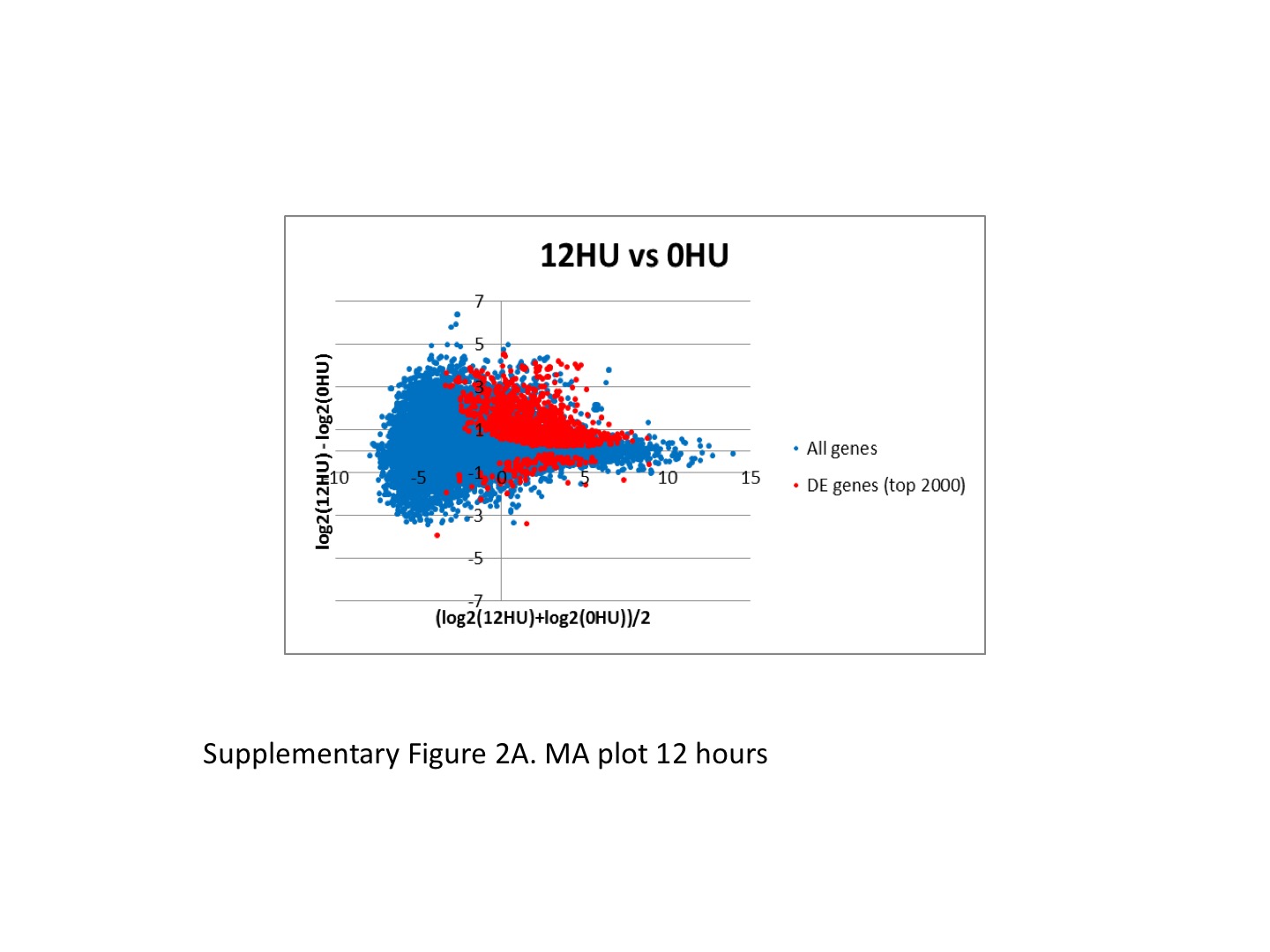

Supplement: Supplementary Figure 2 — (A–G) Tagwise dispersion plots of wound-related transcripts in untreated control mice at specified times after injury. RNA-seq data, expressed as RPKM, was analyzed for differential expression between the 0 h, unwounded, and specific time points afterward. The log2 RPKM gene expression for each transcript (red or blue circle) is ratioed between sample time points to create differential expression as a fold change (Y axis) vs. the absolute level of expression of the transcript (X axis). Transcripts highlighted in red are the top 2,000 most differentially expressed (DE) between the stated time points. [file Image2.JPEG]

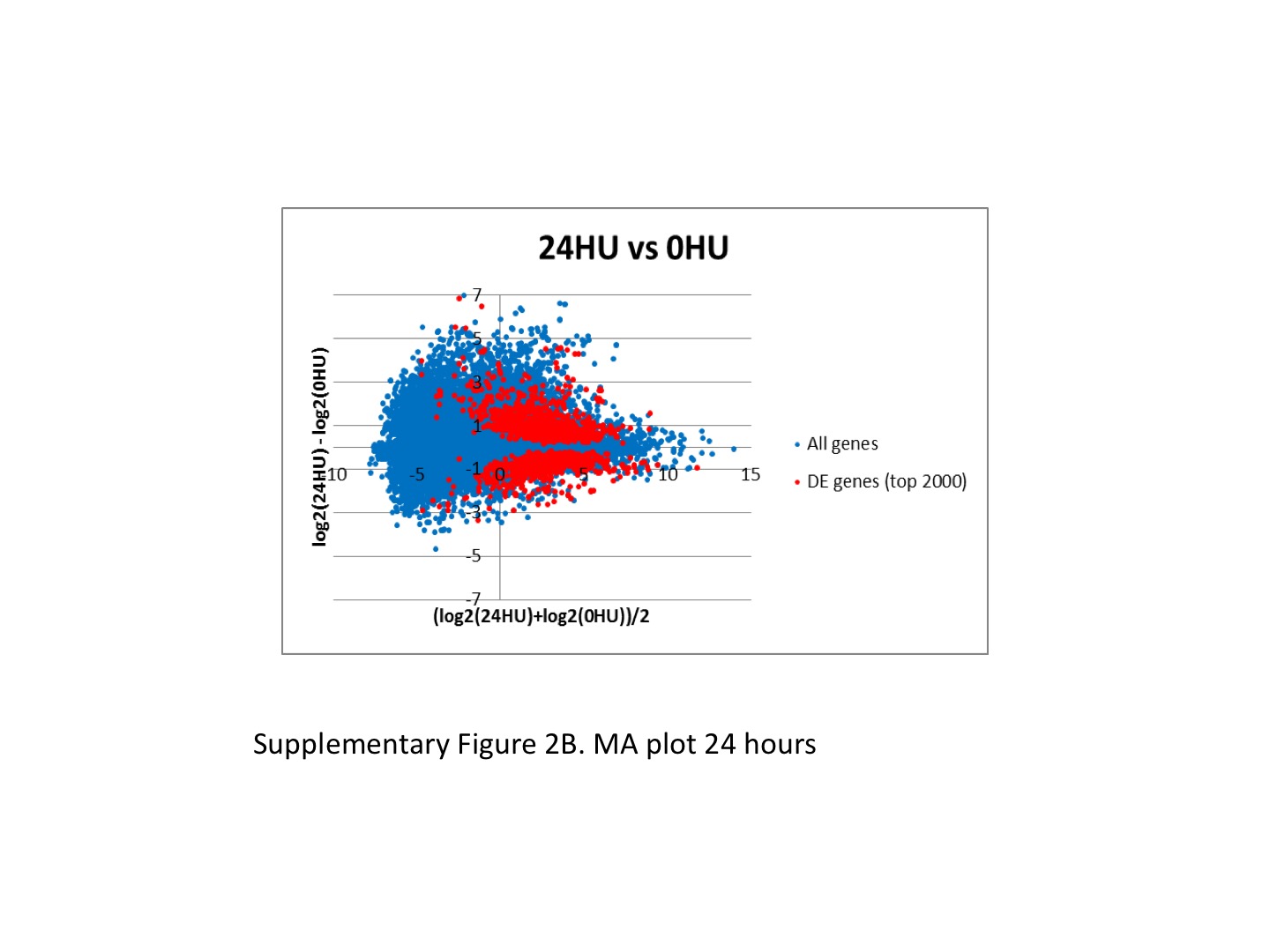

Supplement: Supplementary file 3 [file Image3.JPEG]

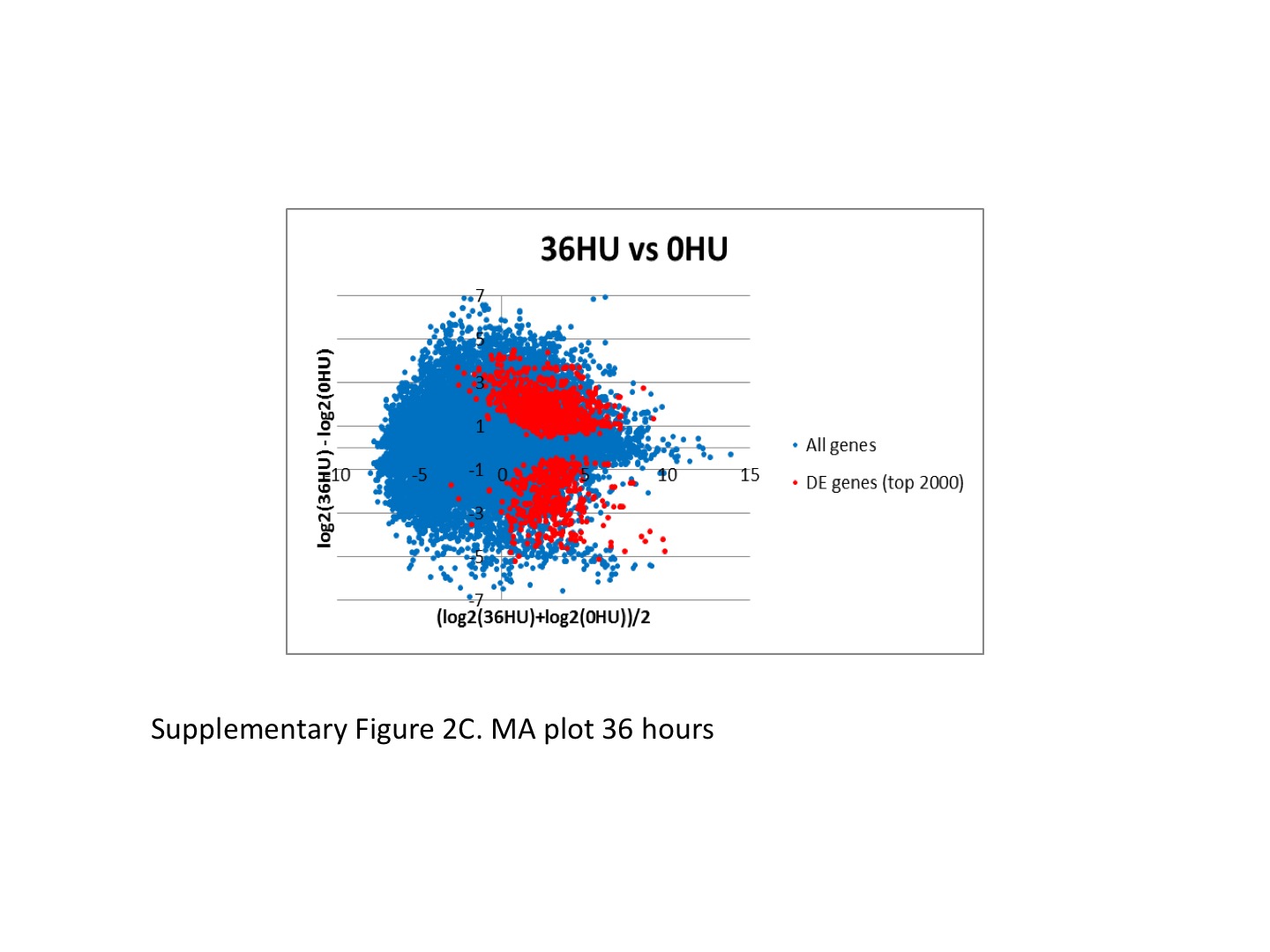

Supplement: Supplementary file 4 [file Image4.JPEG]

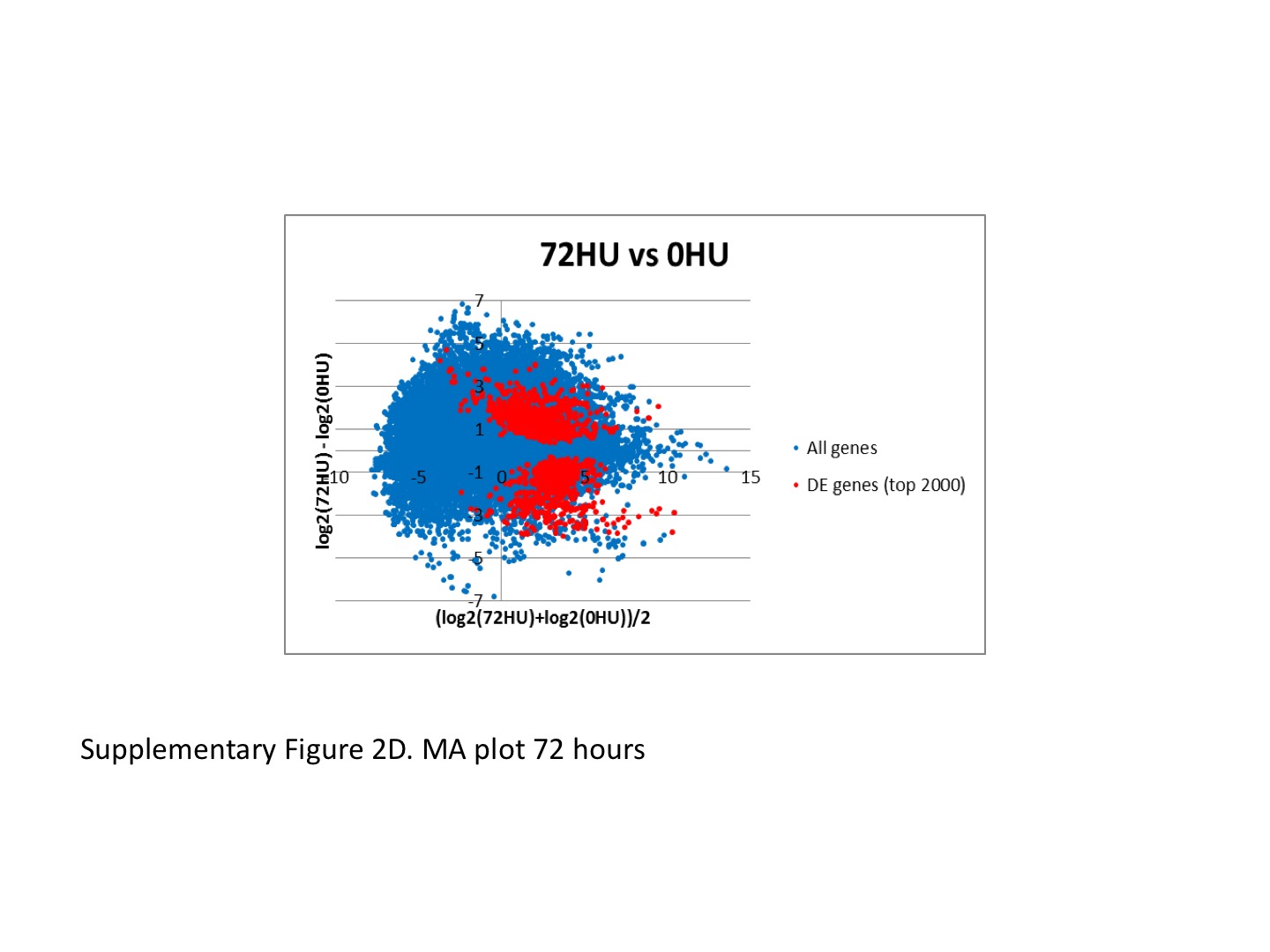

Supplement: Supplementary file 5 [file Image5.JPEG]

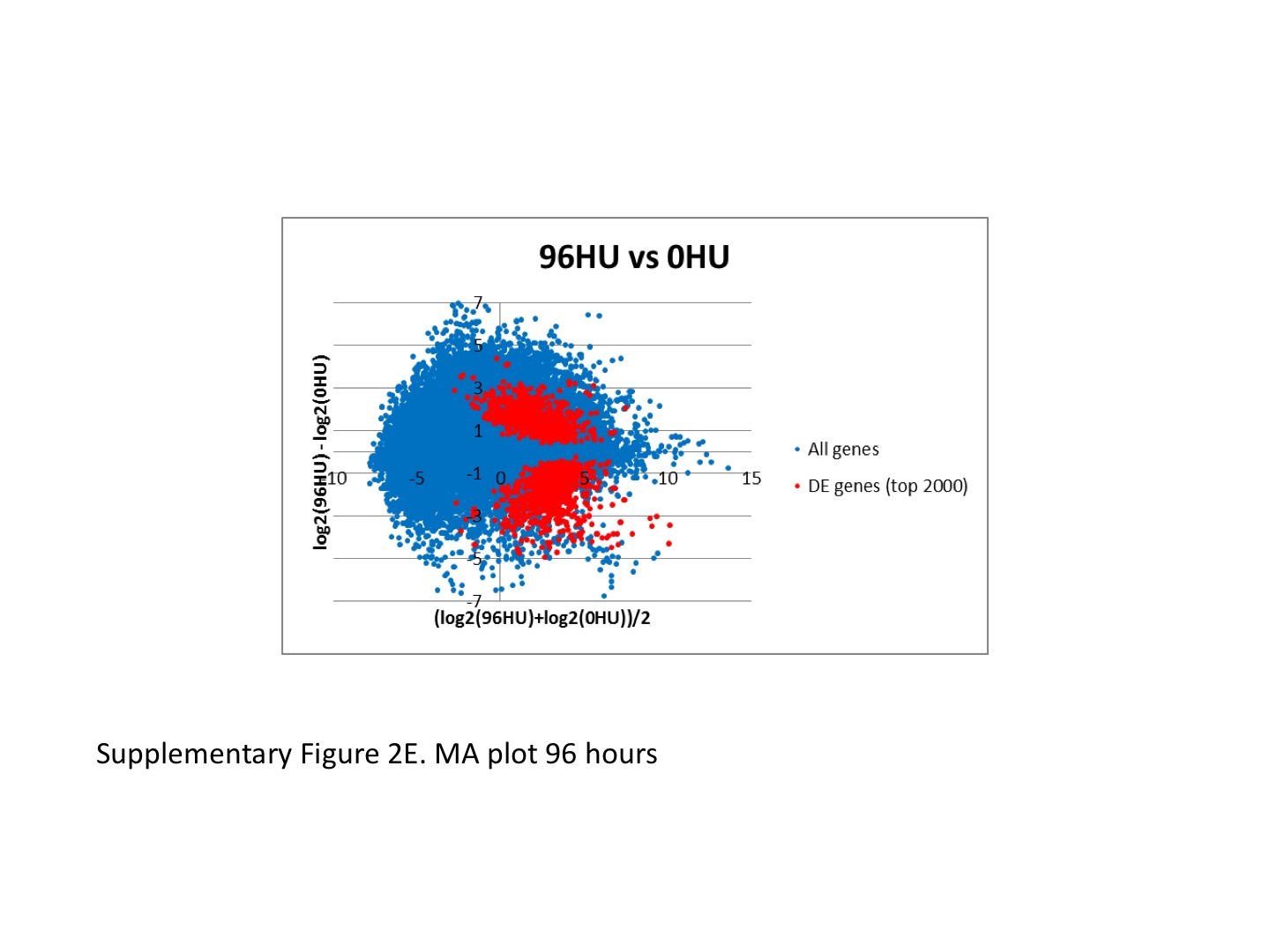

Supplement: Supplementary file 6 [file Image6.JPEG]

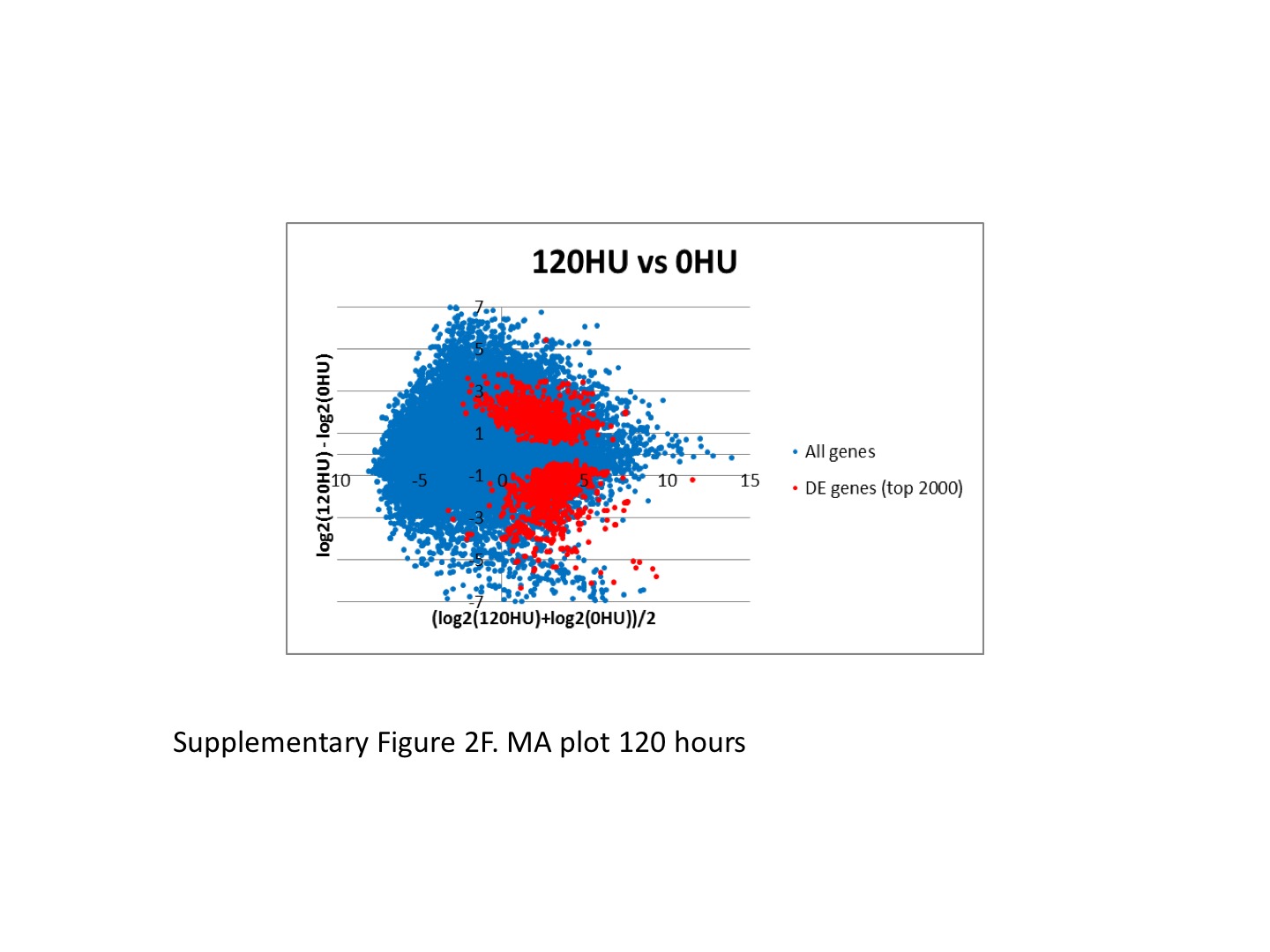

Supplement: Supplementary file 7 [file Image7.JPEG]

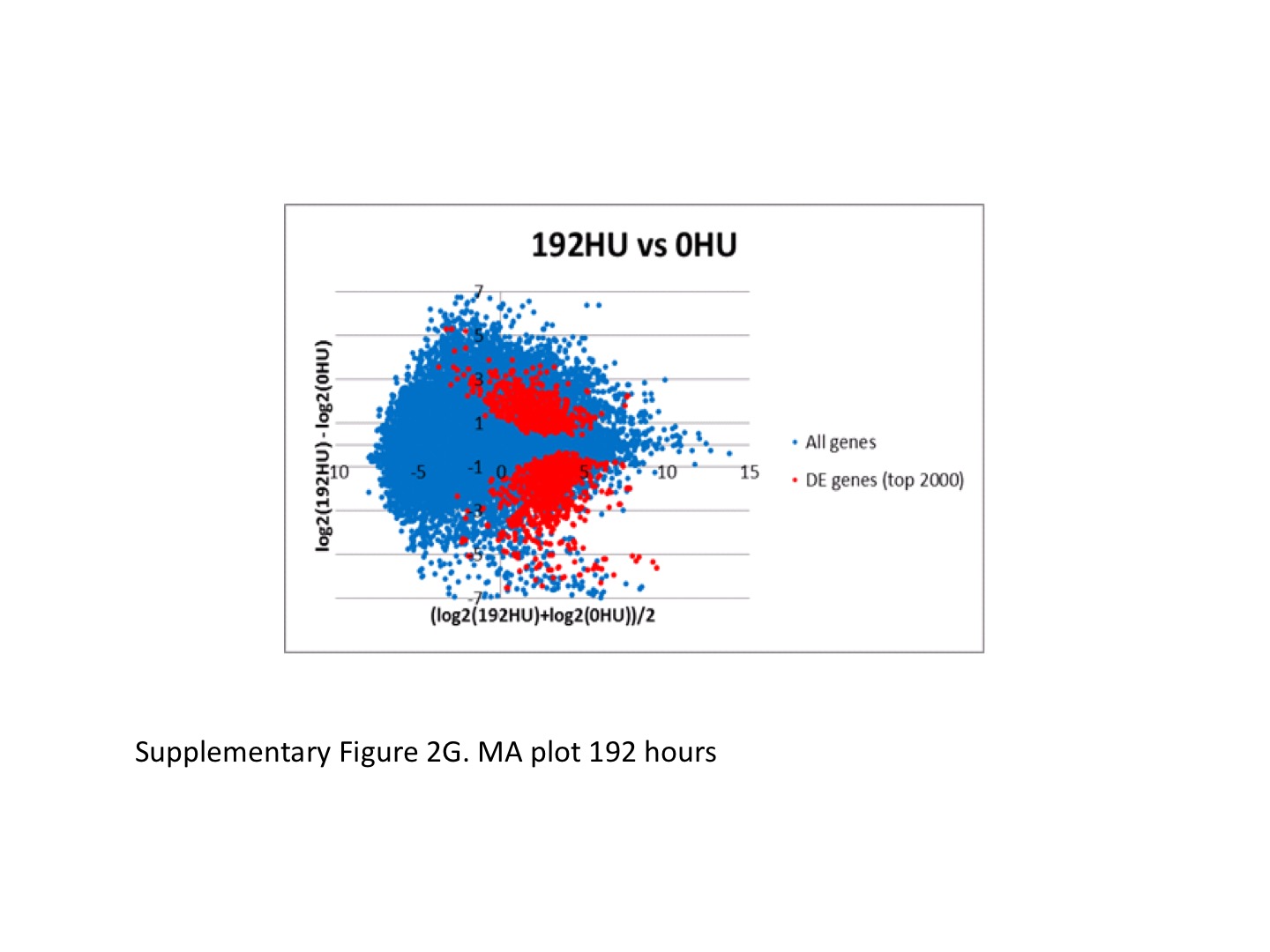

Supplement: Supplementary file 8 [file Image8.JPEG]

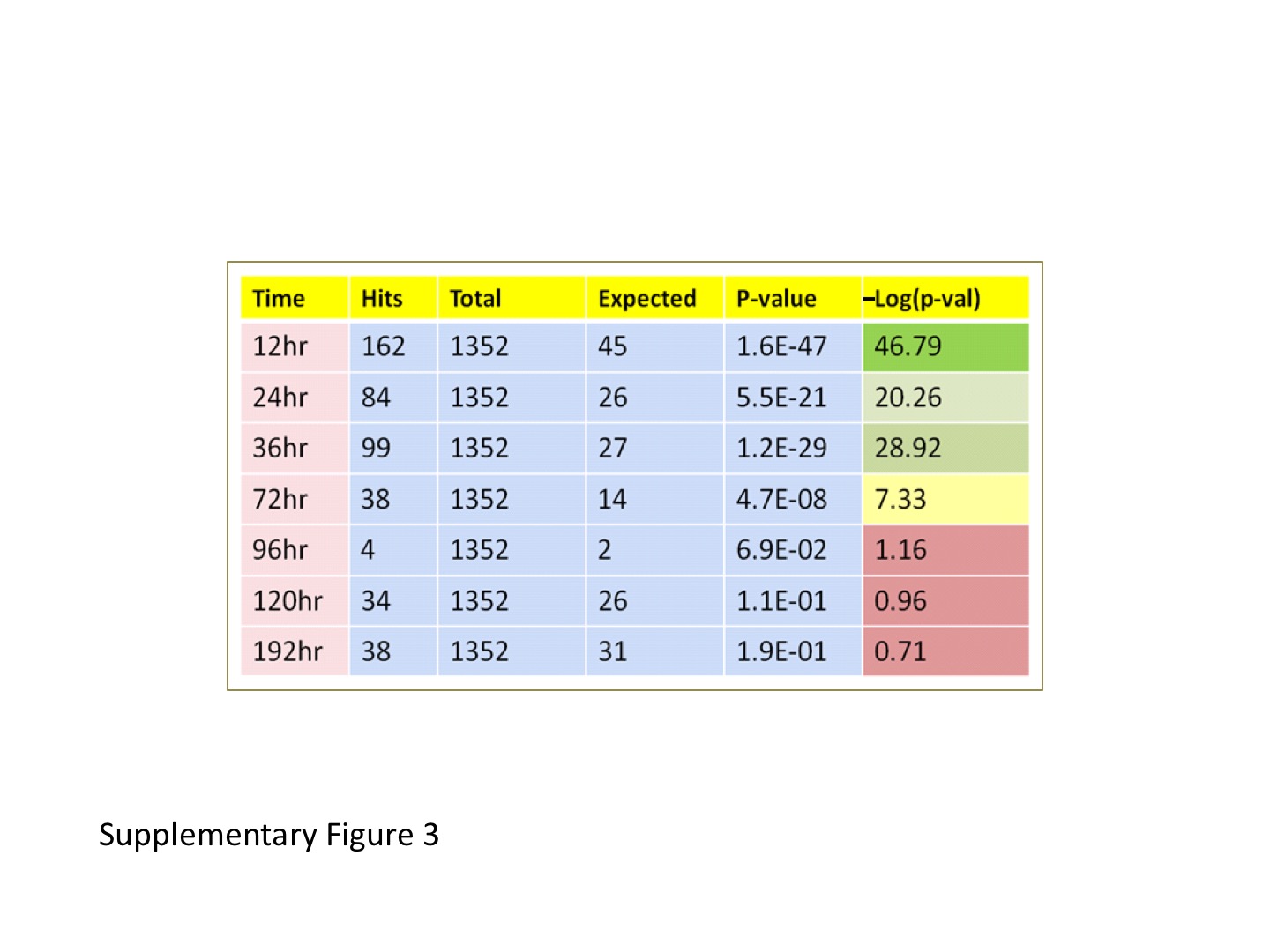

Supplement: Supplementary Figure 3 — Graphic Representation of the intensity of the Response to Wounding GO category at specific time points of the wound healing response. Details of total transcripts affected (hits), total transcripts in pathway, hits expected by chance, and p-value, for the Response to wounding/Wound Healing GO category, at each time point. [file Image10.JPEG]

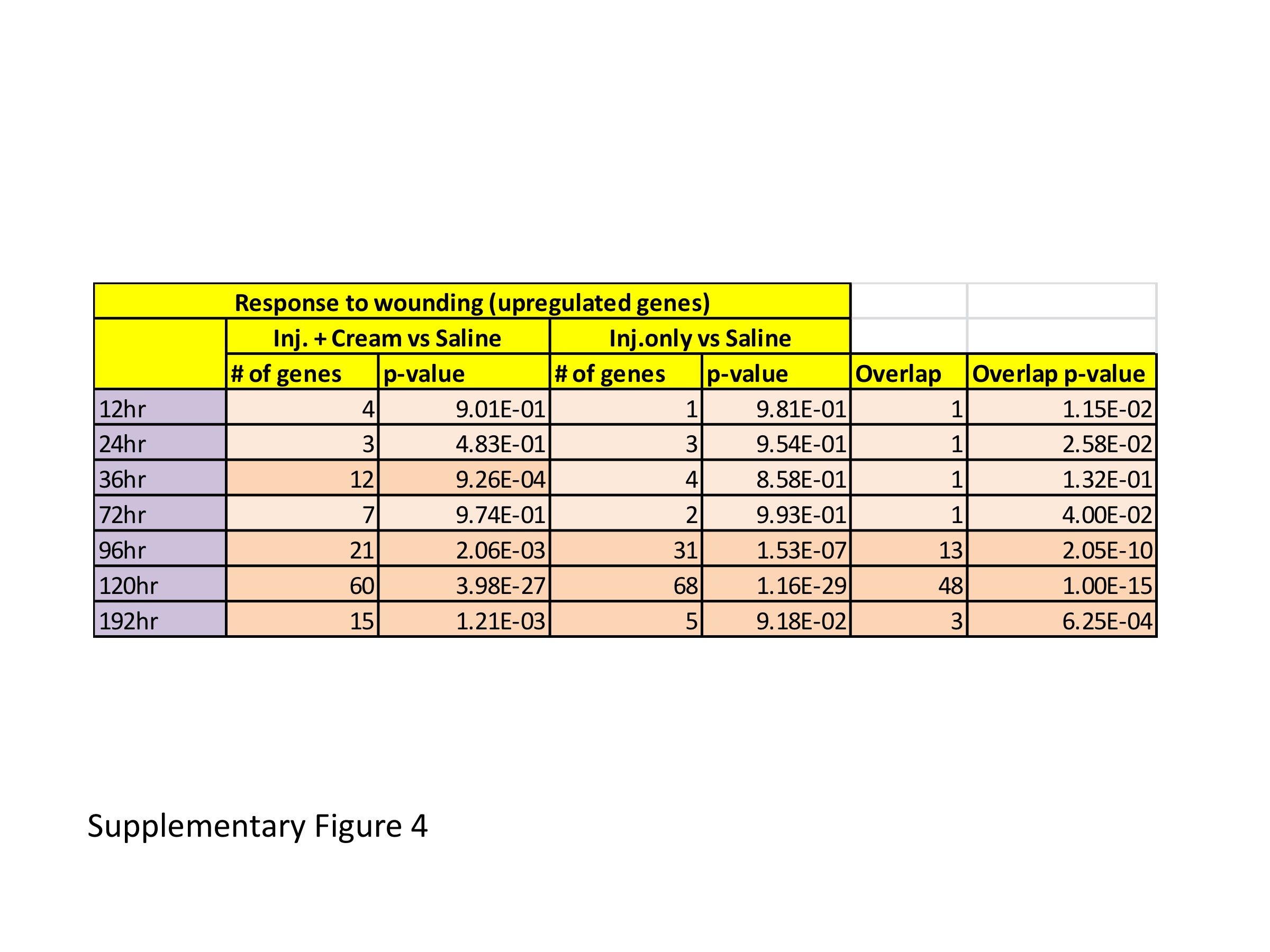

Supplement: Supplementary Figure 4 — The effect of Tr14 treatment on transcripts in the Response to Wounding GO. Upregulated transcripts were calculated between Tr14 vs. saline-treated controls at each of the time points specified as described in Materials and Methods except a p-value threshold of 0.01 was applied. The upregulated transcripts were analyzed for preferential enrichment of the Response to Wounding GO. The two types of Tr14 treatment are shown in separate columns and the overlapping transcripts with Fisher's exact test p-value are counted in the right columns. [file Image11.JPEG]

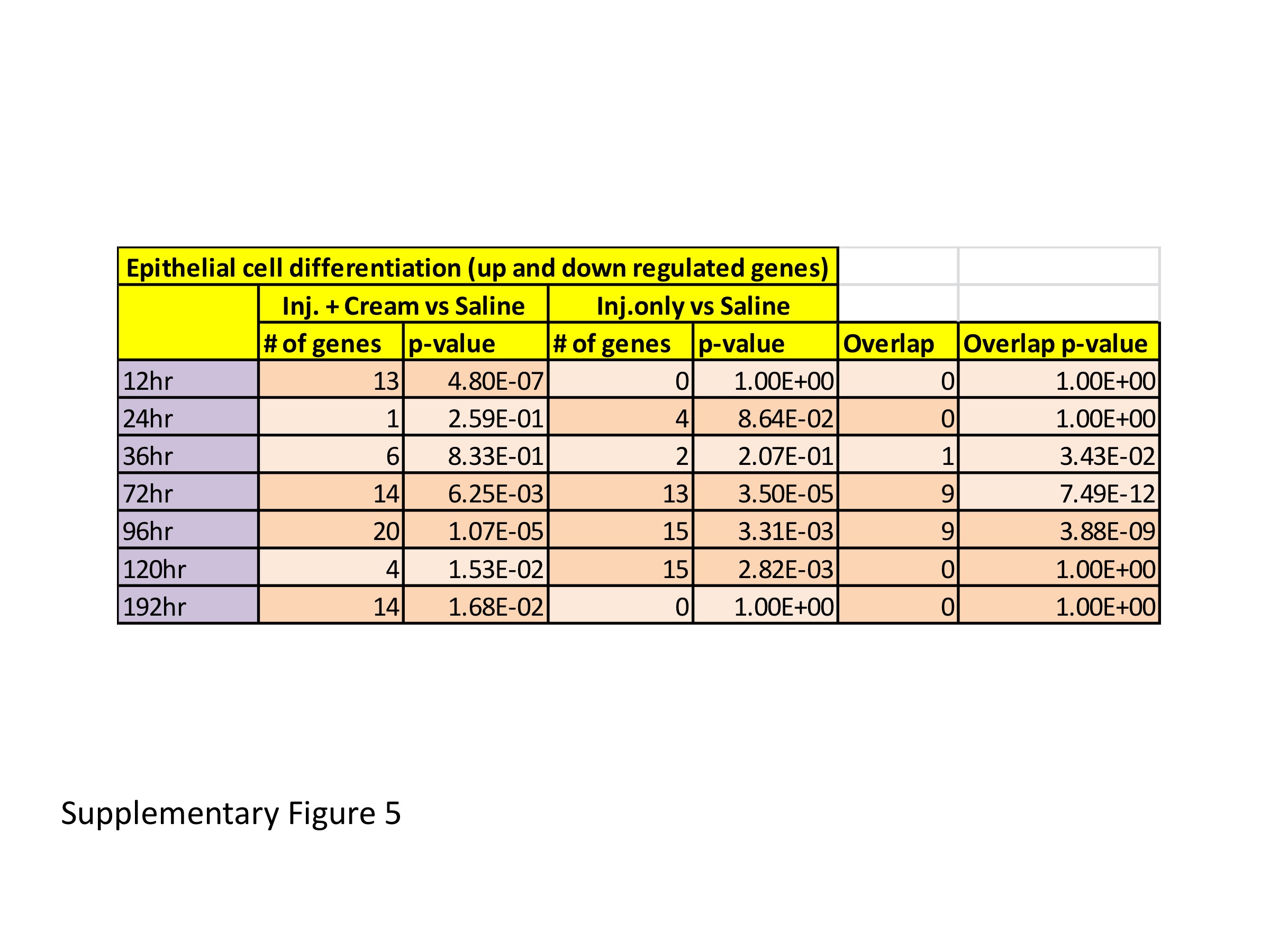

Supplement: Supplementary Figure 5 — The effect of Tr14 treatment on transcripts in the Epithelial Cell Differentiation GO. Details are similar to Supplementary Figure 4 except that both up and down-regulated transcripts in the Epithelial Cell Differentiation GO were analyzed. [file Image12.JPEG]

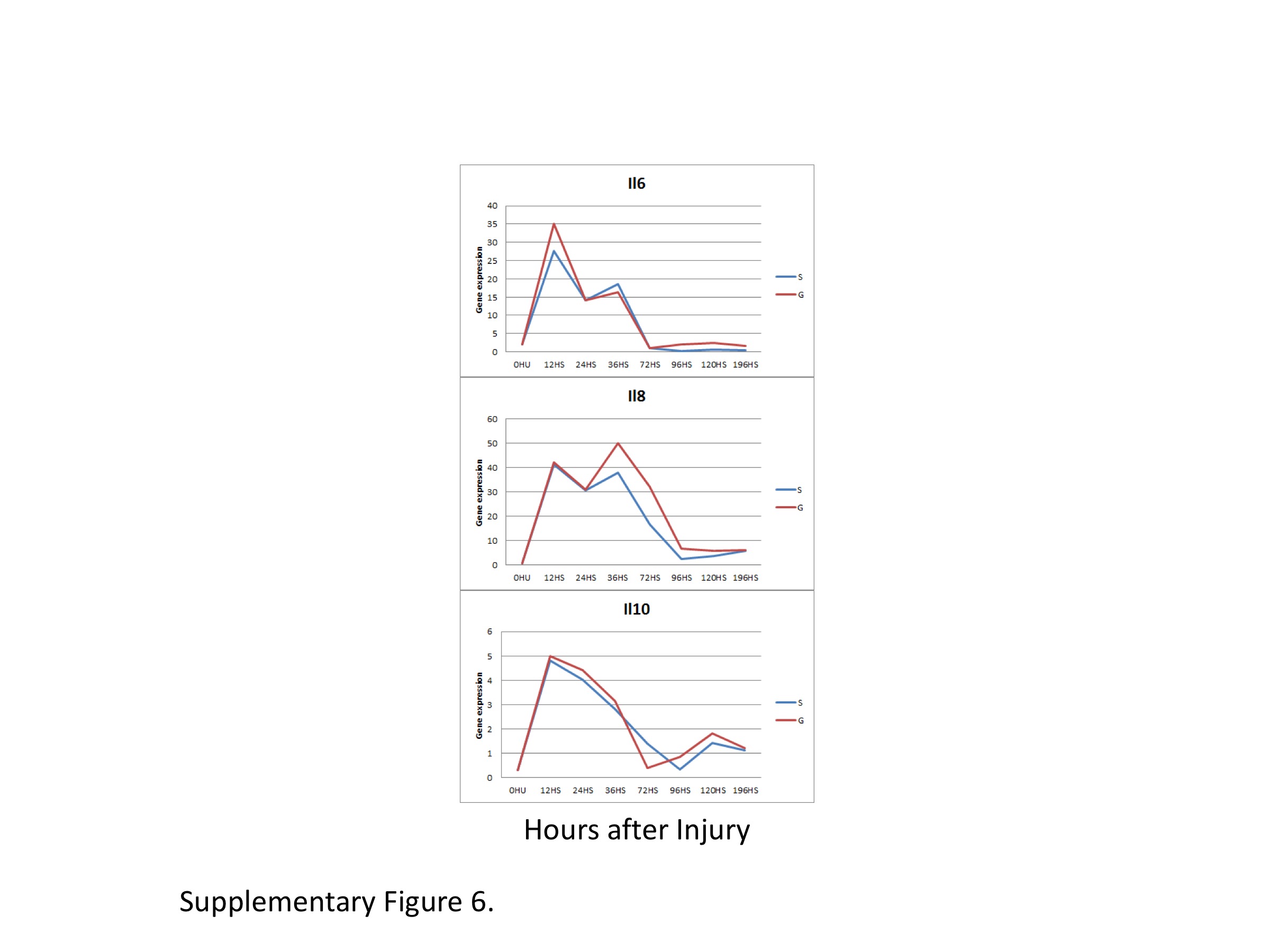

Supplement: Supplementary Figure 6 — The effect of partial-thickness abrasion and Tr14 treatment on IL6, IL8, and IL10 mRNA levels. The levels of 3 well-characterized interleukins is shown over time after injury of the mouse skin. Control wounds treated with saline (S), are compared to wounds treated with Tr14 as a combination of injections and topical treatment as specified (IO, labeled G here). [file Image13.JPEG]
