# Supplementary material for: Deep Sequencing Transcriptome Analysis of Murine Wound Healing: Effects of a Multicomponent, Multitarget Natural Product Therapy-Tr14
Source: Front Mol Biosci. 2017 Aug 17;4:57. doi: 10.3389/fmolb.2017.00057 (PMC5572416; doi:10.3389/fmolb.2017.00057)
Supplement: Supplementary Table 1 — Overall study design, describing the time points, treatment groups, and individual samples sequenced in the study. [file Table1.DOCX]

**Supplementary Table 1: Sample and Sequencing details for the Traumeel Wound Healing Time Course**

| **Time (hrs)**  **Treatment** | | **0hr** | **12hr** | | **24hr** | | **36hr** | | **72hr** | | **96hr** | **120hr** | **192hr** |
| --- | --- | --- | --- | --- | --- | --- | --- | --- | --- | --- | --- | --- | --- |
| **Placebo**  **Treated**  **(U)** | No. of mice | 7 | | 7 | | 7 | | 7 | | 7 | 7 | 7 | 7 |
|  | No. of samples | 14 | | 14 | | 14 | | 14 | | 14 | 14 | 14 | 14 |
|  | No. of samples sequenced | 7 | | 7 | | 7 | | 7 | | 7 | 7 | 7 | 6 |
| **Placebo Treated w/Saline Injection**  **(S)** | No. of mice | N/A | | 7 | | 7 | | 7 | | 7 | 7 | 7 | 7 |
|  | No. of samples | N/A | | 14 | | 14 | | 14 | | 14 | 14 | 14 | 14 |
|  | No. of samples sequenced | N/A | | 6 | | 5 | | 6 | | 6 | 6 | 7 | 7 |
| **Traumeel**  **Injection Treated**  **(Tr14-I)** | No. of mice | N/A | | 7 | | 7 | | 7 | | 7 | 7 | 7 | 7 |
|  | No. of samples | N/A | | 14 | | 14 | | 14 | | 14 | 14 | 14 | 14 |
|  | No. of samples sequenced | N/A | | 6 | | 6 | | 6 | | 6 | 6 | 7 | 7 |
| **Traumeel**  **Injection and Ointment Treated**  **(Tr14-IO)** | No. of mice | N/A | | 7 | | 7 | | 7 | | 7 | 7 | 7 | 7 |
|  | No. of samples | N/A | | 14 | | 14 | | 14 | | 14 | 14 | 14 | 14 |
|  | No. of samples sequenced | N/A | | 6 | | 6 | | 6 | | 6 | 6 | 7 | 7 |
